# Inhibition of asparagine endopeptidase extends the lifespan and improves health span during aging

**DOI:** 10.1016/j.isci.2026.117990

**Published:** 2026-10-23

**Authors:** Menghan Yang, Xin Meng, Zhongyun Xie, Mengmeng Wang, Zhengjiang Qian, Keqiang Ye

**Affiliations:** 1 Brain Cognition and Brain Disease Institute (BCBDI), Shenzhen Institutes of Advanced Technology (SIAT), Chinese Academy of Sciences, Shenzhen, Guangdong 518055, China; 2 University of Chinese Academy of Sciences, Beijing 100049, China; 3 Department of Pharmaceutical Sciences, Faculty of Health Sciences, University of Macau, Taipa, Macau, China; 4 Faculty of Life and Health Sciences, Shenzhen University of Advanced Technology (SUAT), Shenzhen, Guangdong 518107, China

**Keywords:** Asparagine endopeptidase (AEP), legumain, C/EBPβ, senescence, SASP, aging

## Abstract

Asparagine endopeptidase (AEP), also known as legumain, is a lysosomal cysteine protease whose activity increases with age and contributes to age-related tissue dysfunction. We tested whether chronic AEP inhibition could slow aging phenotypes in naturally aged mice. Here, we show that chronic treatment with the AEP inhibitor #11a extended lifespan and improved health span in aged C57BL/6 mice, together with reduced frailty and improved physical and cognitive performance. #11a treatment also attenuated several senescence- and inflammation-associated readouts in multiple organs. Mechanistically, #11a treatment suppressed AEP-dependent cleavage of nicotinamide phosphoribosyltransferase (NAMPT), accompanied by an increased NAD^+^/NADH ratio and higher SIRT1 levels. These findings support further investigation of pharmacological AEP inhibition as a strategy to promote healthy aging.

## Highlights

• #11a extends lifespan in naturally aged wild-type mice
• #11a reduces frailty and improves physical and cognitive performance
• #11a reduces NAMPT cleavage and improves NAD^+^/SIRT1-associated readouts
• #11a attenuates senescence- and inflammation-associated changes across organs

## Graphical abstract

**Figure:**
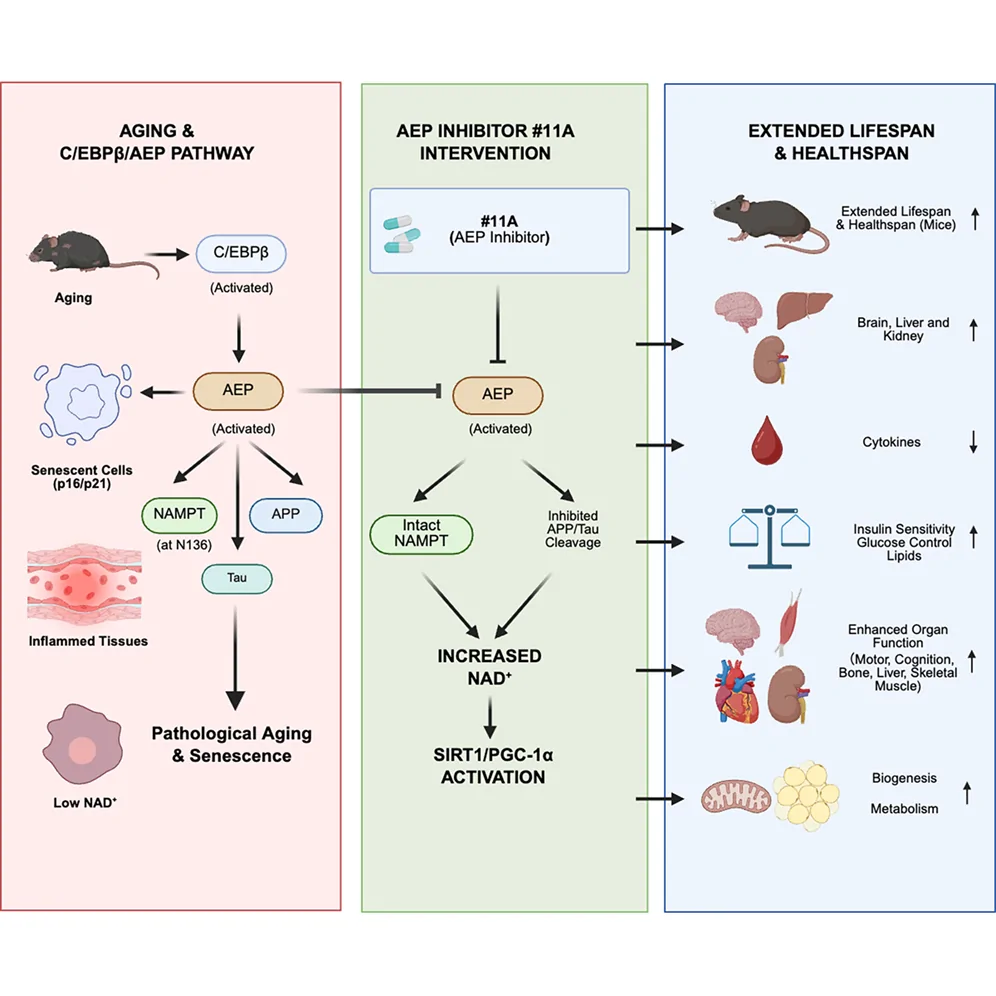

## Teaser

Biological sciences; Biochemistry; Animal physiology

## Introduction

Asparagine endopeptidase (AEP), alternatively referred to as legumain (*LGMN*), is a cysteine protease activated under acidic conditions and belongs to the C13 peptidase family.^1^^,^^2^ AEP is widely distributed, mainly in the kidneys and testes. As an enzyme, AEP specifically cleaves its substrates after asparagine residues and is primarily localized to the endo-lysosomal system^3^; however, under pathophysiological conditions, AEP is also localized in the cytoplasm or in the nucleus.^4^^,^^5^^,^^6^ Increasing evidence links aberrant AEP activation to multiple age-associated pathological processes, particularly in tissues exposed to lysosomal stress and chronic inflammation.^7^^,^^8^^,^^9^ In this state, AEP cleaves a range of target proteins, generating fragmented, often pathogenic, versions that contribute to cellular dysfunction. For instance, we have reported that AEP cleaves Tau N368, producing neurotoxic fragments that aggregate and contribute to neurofibrillary tangle (NFT) formation, a hallmark of Alzheimer’s disease (AD) and brain aging.^10^ Moreover, AEP also processes alpha-synuclein (α-Syn) into truncated forms that are more prone to aggregation, promoting the pathogenesis of Parkinson’s disease (PD).^11^ Furthermore, AEP-mediated cleavage of SET (the protein phosphatase 2A [PP2A] inhibitor, also known as I2PP2A)in neurons impairs the base excision repair pathway and promotes DNA damage accumulation.^12^

Cellular senescence is characterized by the senescence-associated secretory phenotype (SASP), a potent pro-inflammatory secretome that disrupts tissue function and promotes chronic inflammation (“inflammaging”). Several studies have linked AEP activity to neuroinflammatory signaling and related tissue injury.^13^ Aging is also associated with a decline in autophagy, the cell’s recycling process. Dysregulated AEP activity may contribute to this decline by perturbing lysosomal function and proteolytic homeostasis, leading to the accumulation of damaged proteins and organelles.^14^^,^^15^ The deleterious actions of AEP therefore provide a plausible mechanistic link between fundamental aging processes and major age-related diseases, including neurodegenerative diseases, cancers, and cardiovascular disease. For instance, AEP is implicated in AD and PD through its cleavage of Tau and α-Syn.^10^^,^^11^ AEP is often overexpressed in tumors and is involved in processing proteins related to tumor invasion, metastasis, and angiogenesis.^5^^,^^16^^,^^17^ In addition, AEP has emerged as a novel biomarker associated with the development of atherosclerosis^18^^,^^19^ and vascular inflammation.^20^^,^^21^ Most recently, we showed that AEP is augmented in atherosclerosis plaques obtained from patients and proteolytically cuts apolipoprotein A1 (APOA1), impairing cholesterol efflux and high-density lipoprotein (HDL) formation, thereby facilitating atherosclerotic pathologies.^22^ AEP expression is also significantly upregulated in the cortex and hippocampus of wild-type mice subjected to whole-brain irradiation, and genetic ablation of AEP attenuated irradiation-induced neurological deficits in association with reduced neuronal senescence.^23^ Collectively, these observations support testing whether pharmacological AEP inhibition can ameliorate aging-related phenotypes. In the current study, we examined this possibility using the specific inhibitor #11a in naturally aged mice.

## Results

### AEP inhibitor #11a extends the lifespan and improves health span

Age-associated AEP hyperactivation has been linked to multiple pathological processes relevant to aging. We therefore evaluated whether the optimized AEP inhibitor #11a, which displays good oral bioavailability and favorable *in vivo* pharmacokinetic (PK) profiles, could elicit longevity effects in naturally aging C57BL/6 wild-type mice.^11^^,^^24^^,^^25^ Mice were fed a standard chow diet or a diet supplemented with #11a at 0.056 g/kg feed (equivalent to ∼7.5 mg/kg/day) starting at approximately 18 months of age, and lifespan and health span analyses were initiated concurrently. Inhibition of AEP via #11a was associated with longer survival, with mean survival ages of 24 months in the control group and 28 months in the #11a treatment group. The risk of death was significantly higher in the control group than in the #11a group (Mantel-Haenszel hazard ratio [control/#11a] = 2.08, 95% CI 1.02 to 4.27, *p* = 0.0454) (Figure 1A). Although #11a did not affect body weight, food intake was assessed at the cage level (Figures S1A and S1B). During the balanced period of months 1–5 after treatment, in which six cages per group contributed data at every time point, two-way repeated-measures ANOVA showed no significant main effect of treatment (F (1,10) = 2.91, *p* = 0.119) or treatment-by-time interaction (*p* = 0.430). Nevertheless, food intake was numerically lower in #11a-treated cages at several time points. Thus, altered food intake remains a potential contributor to the observed longevity and metabolic phenotypes. We next measured the health span using a frailty index (FI) of 29 phenotypes, scored blindly for severity (0, 0.5, or 1) as indicators of age-related decline in both sexes. Starting at 18 months of age, we conducted repeated assessments every 4 weeks, generating 14 to 15 datasets per group. The overall FI, defined as the average of all phenotypes, served as a metric for morbidity risk (Figure 1B). Because we measured the same mice over time, we used a mixed model that treated each mouse as a random effect and treatment/time as fixed effects. This model was statistically far more efficient than a standard ANOVA (χ^2^, *p* < 0.0001). The analyses showed that blockade of AEP using #11a reduced aging phenotypes and delayed morbidity in both females and males (Figures 1B–1D). Thereafter, we tested the effects of #11a treatment on age-dependent phenotypes utilizing the mixed model (Figures 1E and 1F). #11a significantly decreased the severity of multiple age-dependent phenotypes in females, including mouse grimace, body condition, forelimb grip strength, and hearing loss. Notably, the severity of the movement-related ailments in female mice, including gait disorder and vestibular disturbance, were significantly reduced by #11a (Figure 1E). In males, the severity of mouse grimace, cataract, loss of fur color, tail stiffening, menace reflex, rectal prolapse, and diarrhea was decreased for all of these phenotypes (Figure 1F).

**Figure 1:**
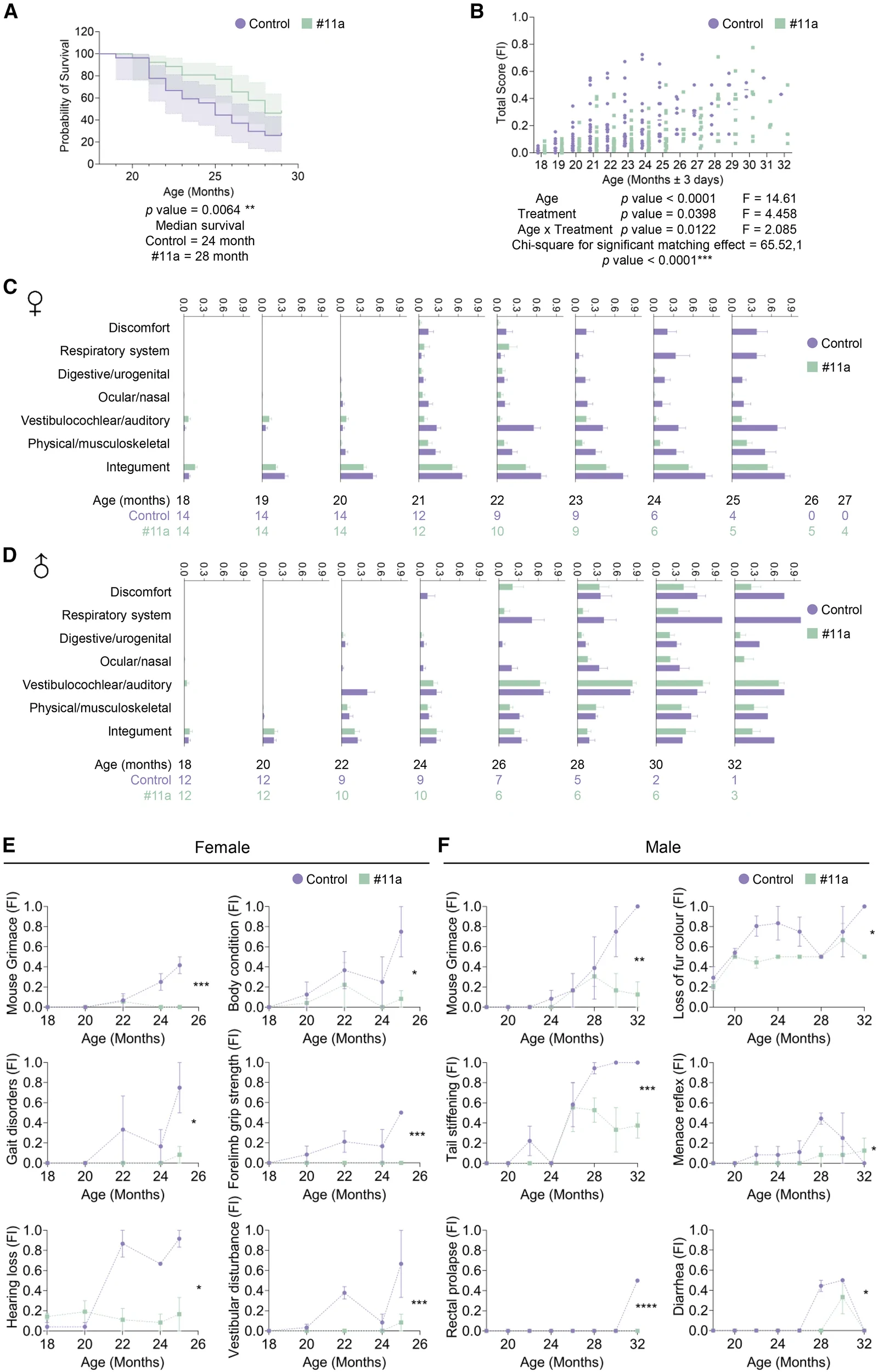
#11a extends the lifespan and improves health span (A) Kaplan-Meier survival curves of control (*n* = 26) and #11a-fed mice (*n* = 26). Statistical comparison was performed using the log rank test. The shaded area denotes the 95% confidence band of the fitted curve. (B) Total frailty index (FI) scores during lifespan, comparing control mice (purple) to those fed #11a in the diet (green) starting at 18 months of age. Each point represents an individual animal; *n* represents all animals alive at each measurement time. A mixed model was used to analyze the data longitudinally. A linear mixed-effects model was applied with mouse ID as a random effect and treatment and time as fixed effects. The mixed model fit was compared with a simple ANOVA model using a chi-square test. (C and D) Individually graphed frailty phenotypes that significantly increased with aging, comparing control with #11a-fed mice for (C) female and (D) male cohorts. The number of animals assessed at each time point is shown as row beneath each graph. (E and F) Trajectories for age-dependent frailty phenotypes for (E) female and (F) male mice in the study. A mixed model was used to evaluate the effect of #11a on each phenotype. Data are presented as mean ± SEM unless otherwise indicated. Significance levels are indicated as follows: ∗*p* < 0.05; ∗∗*p* < 0.01; ∗∗∗*p* < 0.001; ∗∗∗∗*p* < 0.0001.

Among the frailty measures influenced by #11a, age-related vision loss and coat condition showed the most notable improvement. Histological analysis using hematoxylin and eosin (H&E) staining demonstrated that #11a treatment was associated with improved lens structure and a lower incidence of cataracts in aged mice. This was accompanied by increased cell densities in both the inner nuclear layer (INL) and outer nuclear layer (ONL) of the retina (Figure S1C).

The plasma concentrations of #11a were measured by liquid chromatography-tandem mass spectrometry (LC-MS/MS) (Figure S2A). Following 5 months of treatment, histopathological assessment of major organs, including the heart, liver, spleen, lungs, and kidneys, revealed no overt abnormalities in the examined tissues at this intermediate time point (Figure S2B). Consistent with this, complete blood count (CBC) and serum biochemical analyses did not reveal major abnormalities in the assessed parameters (Table S1).

### Inhibition of AEP alleviates the physical and cognitive decline during aging

To examine the effect of #11a on age-related physical and cognitive decline, behavioral assessments were performed in 23-month-old C57BL/6 mice. We found that inhibition of AEP for 5 months significantly enhanced falling time and elevated falling speed, concomitant with a trend toward longer retention on the treadmill belt as compared to vehicle control (Figure 2A). The wire hanging assay showed that #11a substantially increased the hanging time on the pole versus the control (Figure 2B). In the open-field test, #11a-treated mice showed altered exploratory behavior, including increased total distance moved, increased center exploration, and reduced immobility time, although these findings should be interpreted cautiously because the current dataset does not fully distinguish anxiety-related behavior from general locomotor changes (Figures 2C and 2E). Behavioral testing at 23 months of age also supported improved performance in the novel object recognition (NOR) test (discrimination index: 32% ± 15.69% vs 48% ± 11.03%; *p* = 0.0071, *n* = 12) and Y maze tests in #11a-treated mice compared with vehicle controls (exploration times: 68 s ± 20.85 vs. 102 s ± 20.35; *p* = 0.0005, *n* = 12) (Figures 2C–2H).

**Figure 2:**
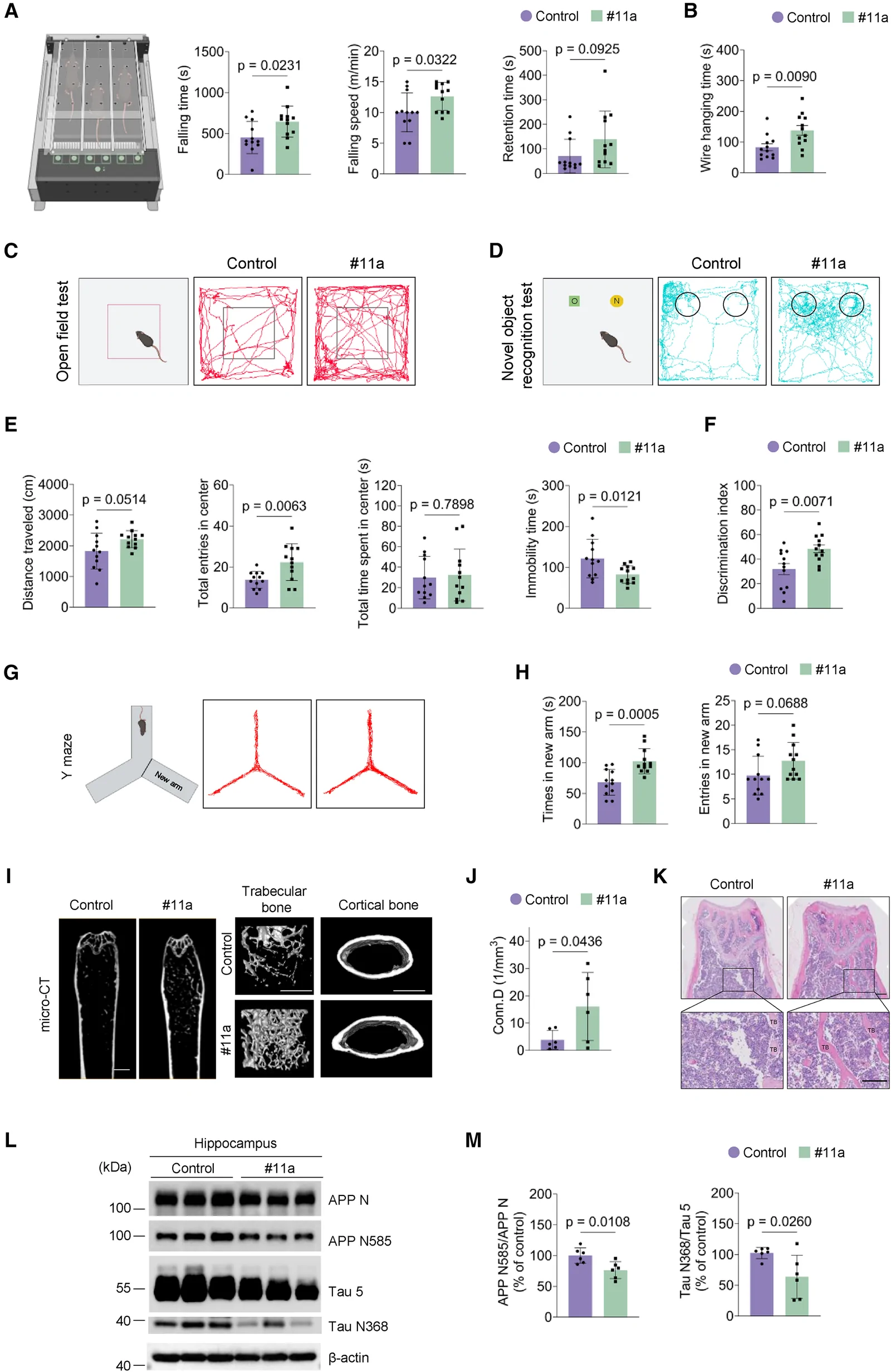
#11a ameliorates age-related decline in physical and cognitive functions (A) Treadmill performance of control and #11a-treated mice. Average falling time (left) and average of the maximum falling speed (middle) were obtained from exhaustive tests, and retention time (right) from the endurance test (*n* = 12). (B) Wire hang test performance (*n* = 12). (C) Representative traces from the open-field test. (D) Representative traces from the novel object recognition test. (E) Behavioral parameters in the open-field test: distance traveled, total entries into the center, total time spent in the center, and immobility time (*n* = 12). (F) Novel object recognition test for the assessment of recognition memory (*n* = 12). (G) Representative traces from the Y maze test. (H) Y maze test for the evaluation of spatial working memory (*n* = 12). (I) Representative micro-CT images of femoral bone structures, including trabecular and cortical bone, collected at 23 months of age. Scale bars: 1.0 mm. (J) Micro-CT measurements of connectivity density (Conn.D). (K) Representative H&E staining images of femurs (upper) and a higher-magnification view of the boxed region (lower). Scale bars: 200 μm. (L and M) Western blot analysis (L) and quantification (M) of APP, APP N585, Tau 5, and Tau N368 protein levels in the hippocampus (*n* = 6). Data are presented as mean ± SD. For behavioral tests, *n* = 12 per group; for micro-CT, histological, and immunoblotting analyses, *n* = 6 per group unless otherwise indicated. Statistical significance was determined by unpaired two-tailed Student’s *t* test. Significance levels are indicated as ∗*p* < 0.05, ∗∗*p* < 0.01, ∗∗∗*p* < 0.001, ∗∗∗∗*p* < 0.0001.

Micro-computed tomography (micro-CT) analysis of femurs harvested at sacrifice showed that connectivity density (Conn.D) was significantly increased in #11a-treated mice (Figure 2J), whereas most other trabecular and cortical parameters were not significantly altered (Figure S3). However, #11a did not significantly alter other trabecular bone microarchitecture parameters, including bone surface (BS), bone volume fraction (BV/TV), total volume (TV), bone surface-to-bone volume ratio (BS/BV), bone surface-to-total volume ratio (BS/TV), structural model index (SMI), trabecular spacing (Tb.Sp), trabecular number (Tb.N), and trabecular thickness (Tb.Th) (Figure S3A). Moreover, the cortical bone morphological parameters, including cortical thickness (Ct.Th), cortical bone area (Ct.Ar), and the ratio of cortical bone area to total tissue area (Ct.Ar/Tt.Ar), were not significantly changed either by #11a chronic treatment (Figure S3B). To further investigate the skeletal phenotype associated with #11a during aging, we performed H&E staining and analyzed the bone morphology and white adipocytes. We observed that white adipocytes were reduced and trabecular bone was increased in the bone from #11a-treated mice as compared to vehicle control (Figure 2K).

Our previous studies show that AEP cleaves amyloid precursor protein (APP) at N585 and stimulates Aβ production, and it also cleaves Tau at N368 to trigger its hyperphosphorylation and aggregation.^10^^,^^26^ Accordingly, we conducted immunoblotting analysis and found that #11a blocked AEP fragmentation of APP N585 and Tau N368 (Figures 2L and 2M). These findings are consistent with the improved cognitive performance observed in the NOR and Y maze tests after AEP inhibition by #11a. Together, these data support an association between #11a treatment and attenuated age-related decline in physical and cognitive function.

### Blockade of AEP diminishes NAMPT fragmentation and elevates SIRT1 levels

Nicotinamide adenine dinucleotide (NAD^+^) is an essential enzymatic cofactor and co-substrate in numerous critical biological pathways. Nicotinamide phosphoribosyltransferase (NAMPT) is the rate-limiting enzyme that catalyzes NAD^+^ biosynthesis from nicotinamide.^27^ Most recently, we reported that AEP cleaves NAMPT at N136 and depletes NAD^+^, triggering neuronal senescence and cognitive deficits. Knockout of AEP or blockade of NAMPT cleavage by AEP attenuates these defects in neuronal Thy 1-C/EBPβ transgenic (Tg) mice.^28^ To examine whether AEP inhibitor #11a improves cognitive functions by interfering with NAMPT fragmentation by AEP and delaying brain aging, we performed immunoblotting with the hippocampal tissues and found that #11a pronouncedly reduced active AEP levels. Consequently, NAMPT N136 fragmentation by active AEP was reduced, which was validated by the C137-specific antibody that recognized the truncated but not the full-length NAMPT. Sirtuins are an evolutionarily conserved family of NAD^+^-dependent deacetylases and play a critical part in aging and longevity control in diverse model organisms.^29^^,^^30^ The ubiquitously expressed transcriptional co-activator peroxisome proliferator-activated receptor gamma-coactivator 1 alpha (PGC-1α) acts as a master regulator of mitochondrial function and is highly expressed in the brain, mediating brain aging.^31^ PGC-1α is a downstream target of SIRT1.^32^ Subsequently SIRT1 and PGC-1α levels were elevated by #11a (Figure 3A). To assess whether major NAD^+^-consuming pathways other than SIRT1 contributed to these observations, we evaluated CD38 protein abundance, CD38 NADase activity, and global poly(ADP-ribose) (PAR) levels as a PARP pathway readout. CD38 protein levels were not significantly altered by #11a treatment in hippocampal or quadriceps tissues (Figures S5A and S5B). Recombinant human CD38 exhibited robust ε-NAD hydrolysis that was suppressed by the CD38 inhibitor 78c, confirming assay responsiveness. In hippocampal and quadriceps tissue lysates, inclusion of 78c reduced the NADase signal, indicating that a substantial fraction of the measured ε-NAD hydrolysis was CD38-dependent. Under these assay conditions, CD38 NADase activity was not significantly altered by #11a treatment (Figures S5C and S5D). In parallel, as a technical positive control, H_2_O_2_ increased PARylation in HT22 cells, and olaparib reduced this induction, validating the PAR readout (Figures S5E and S5F).^33^ In brain and quadriceps tissues, #11a did not significantly alter PARP1 protein abundance or global PAR levels under the tested conditions (Figures S5G and S5H). These findings do not establish direct target engagement of CD38 or PARP by #11a. Immunofluorescent (IF) co-staining with antibodies against total AEP and active AEP showed that active AEP fluorescent intensities relative to total AEP in the hippocampal neurons in both CA1 and CA3 regions were significantly decreased (Figure 3B). Quantification of both immunoblotting analysis and IF images confirmed these observations (Figures 3C and 3D). Moreover, an AEP enzymatic assay further corroborated that #11a potently inhibited AEP activity in the brain (Figure 3E). We also conducted similar biochemical assays with quadriceps tissues and found that active AEP and NAMPT C137 cleavage in muscle were also inhibited by #11a, associated with higher SIRT1 and PGC-1α levels (Figures 3F and 3G), together with reduced α-smooth muscle actin (α-SMA), a marker of fibrotic progression (Figure 3H). In addition, #11a significantly increased the NAD^+^/NADH ratio in brain, muscle, liver, and kidney tissues, which is consistent with the idea that inhibition of AEP-mediated NAMPT cleavage may help preserve NAD homeostasis (Figure 3I, see also NAD^+^ and NADH levels in Figure S4). Together, these results show that #11a inhibits AEP activity, reduces NAMPT fragmentation, and is associated with increased SIRT1 and PGC-1α levels in both brain and muscle.

**Figure 3:**
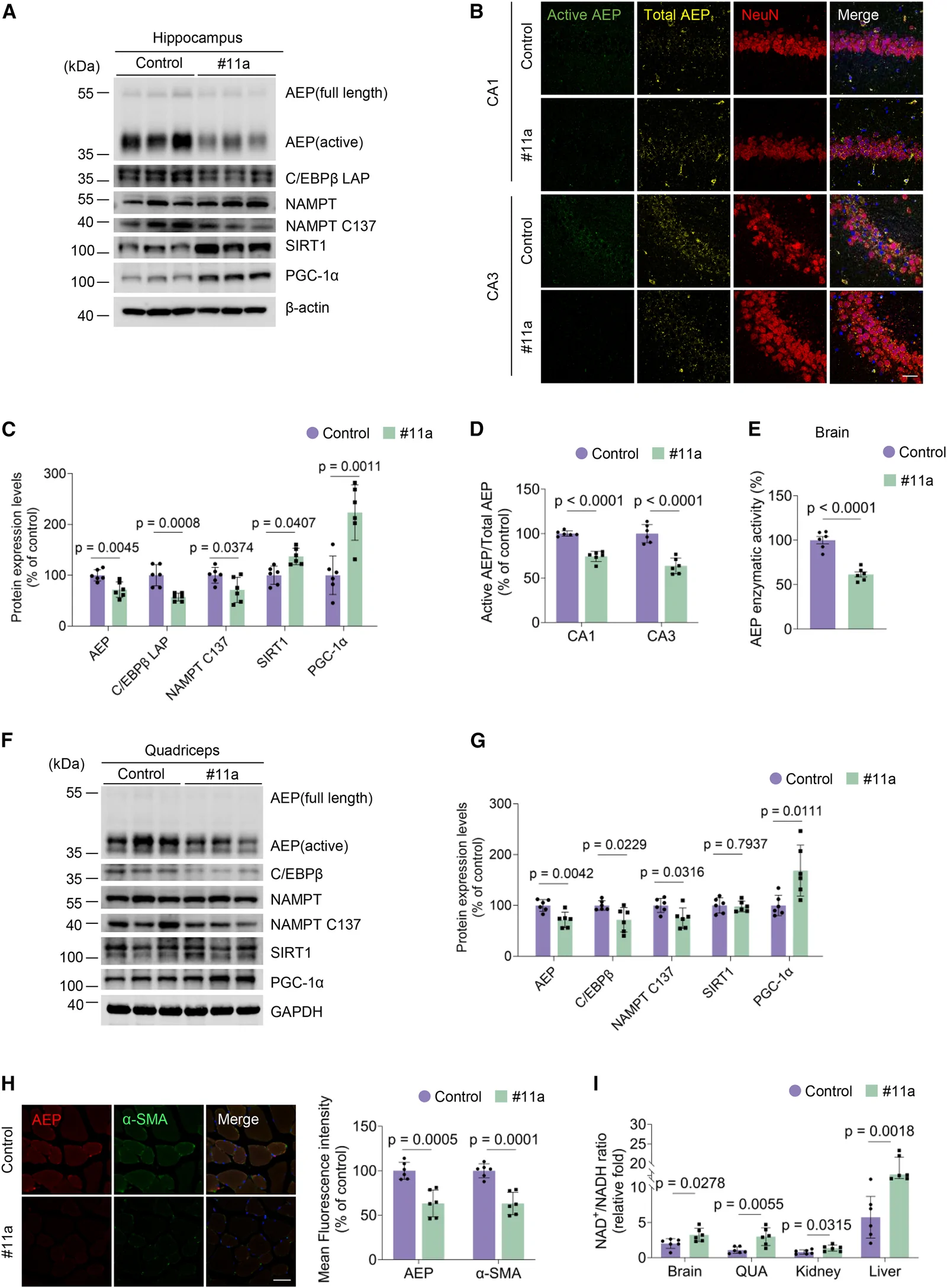
#11a modulates aging-related pathways in the tissues from aged mice (A and C) Western blot analysis (A) and quantification (C) of AEP, C/EBPβ LAP, NAMPT, NAMPT C137, SIRT1, and PGC-1α protein levels in the hippocampus of 23-month-old mice (*n* = 6). (B and D) Immunofluorescence staining (B) and quantification (D) of total AEP, active AEP, and NeuN in the hippocampus (*n* = 6). Scale bars: 50 μm. (E) AEP enzymatic activity measured in hippocampal tissue lysates (*n* = 6). (F and G) Western blot (F) and quantification (G) of AEP, C/EBPβ LAP, NAMPT, NAMPT C137, SIRT1, and PGC-1α protein expression in the quadriceps muscle (*n* = 6). (H) Immunofluorescence staining and quantification of AEP and α-smooth muscle actin (α-SMA) in the quadriceps sections (*n* = 6). Scale bars: 50 μm. (I) NAD^+^/NADH ratio measured in the brain, quadriceps muscle, liver, and kidney tissues (*n* = 6). Data are presented as mean ± SD. Statistical significance was determined by unpaired two-tailed Student’s *t* test. Significance levels are indicated as ∗*p* < 0.05, ∗∗*p* < 0.01, ∗∗∗*p* < 0.001, ∗∗∗∗*p* < 0.0001.

### Suppression of AEP is associated with improved metabolic and adipose-associated readouts during aging

The decline in insulin sensitivity (insulin resistance) and altered adipose remodeling are hallmark metabolic features of aging. These processes are deeply interconnected, creating a vicious cycle that drives age-related metabolic dysfunction and increases the risk for type 2 diabetes, cardiovascular disease, and frailty. Notably, both the glucose tolerance test (GTT) and insulin tolerance test (ITT) showed that #11a treatment significantly increased insulin sensitivity and reduced blood glucose concentrations compared with vehicle control (Figures 4A and 4B). In addition, alanine aminotransferase (ALT), aspartate aminotransferase (AST), low-density lipoprotein cholesterol (LDL-C), and lactate dehydrogenase (LDH) concentrations in the plasma were significantly decreased (Figure 4C). Histological analysis showed that #11a substantially reduced adipocyte accumulation in brown adipose tissue (BAT) and inguinal white adipose tissue (iWAT), which was further validated by Oil Red O staining. With the reduction of adipocyte cells, tibialis anterior muscle (TA) fiber diameters were significantly augmented (Figures 4D and 4E). BAT and iWAT also produce adipokines and participate in age-related inflammatory remodeling. Quantitative reverse-transcription PCR (RT-qPCR) analysis revealed that several key genes implicated in glucose or fatty acid metabolism, including *Adipoq* (adiponectin), *Glut4* and *Pparg* in BAT and *Pgc1a*, *Ucp1* and *Dio2* in iWAT, were substantially elevated after chronic #11a treatment (Figures 4F and 4G).

**Figure 4:**
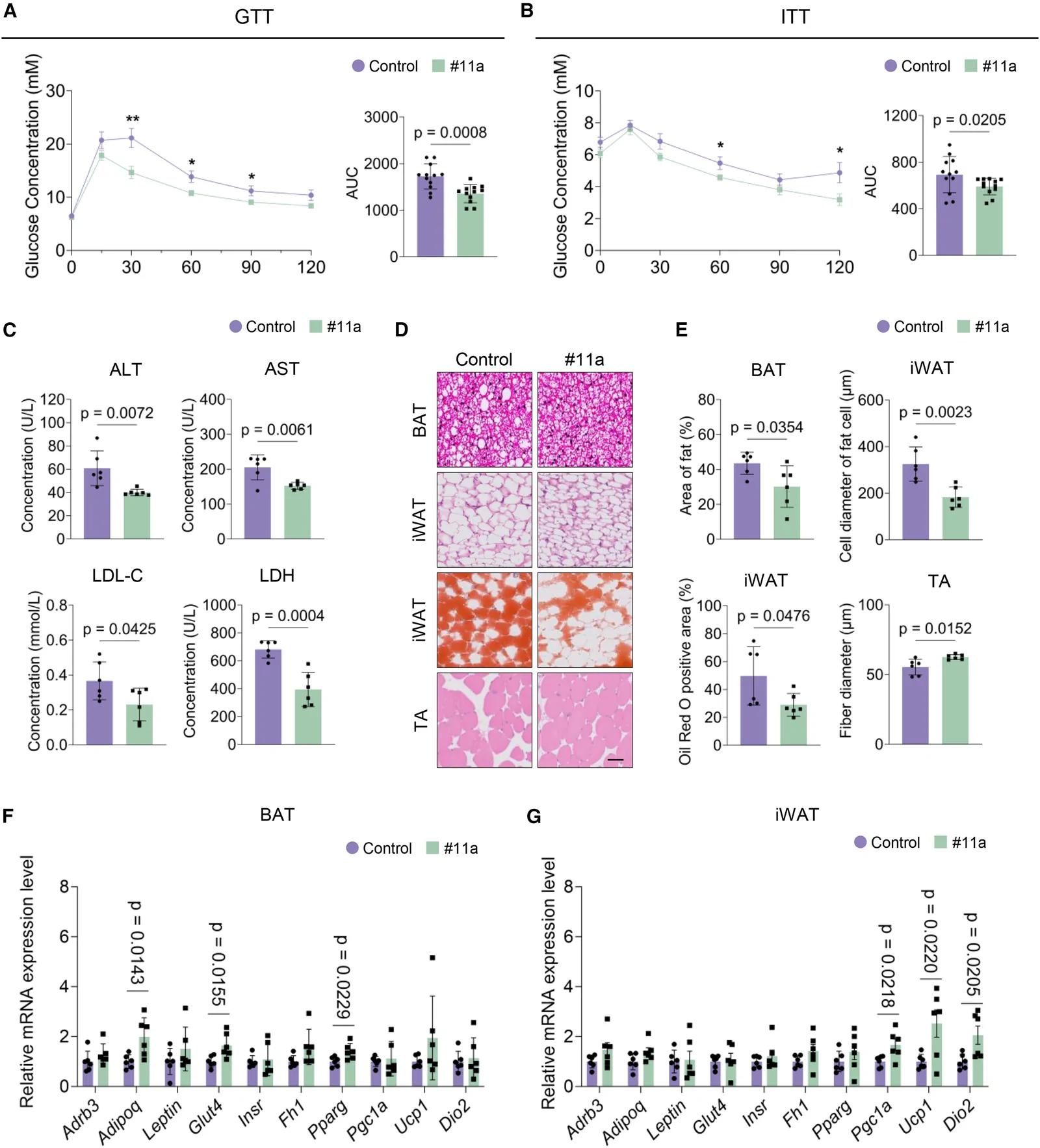
#11a is associated with improvements in selected metabolic and adipose-associated readouts (A) Left: Blood glucose levels (mM) during the GTT after intraperitoneal injection of glucose (2 g/kg body weight). The shaded area denotes the 95% confidence band of the fitted curve. Right: area under the curve (AUC) for the GTT (*n* = 12). (B) Left: Blood glucose levels (mM) during the ITT after intraperitoneal injection of insulin (0.5 U/kg body weight). The shaded area denotes the 95% confidence band of the fitted curve. Right: AUC values for the ITT (*n* = 12). (C) Plasma concentrations of alanine aminotransferase (ALT), aspartate aminotransferase (AST), low-density lipoprotein cholesterol (LDL-C), and lactate dehydrogenase (LDH), measured using standard automated biochemical assays (*n* = 6). (D) Representative histological images showing H&E staining of inguinal white adipose tissue (iWAT), brown adipose tissue (BAT), tibialis anterior muscle (TA), and Oil Red O staining of iWAT. Scale bars: 50 μm. (E) Quantification of adipocyte area in iWAT and BAT, and of TA fiber cross-sectional area, from (D) (*n* = 6). (F and G) Quantitative real-time PCR (real-time qPCR) analysis of key metabolic genes in (F) BAT and (G) iWAT (*n* = 6). Gene expression was normalized to Gapdh and is presented as fold-change relative to the control group. Data are presented as mean ± SD. AUC and other endpoint comparisons used unpaired two-tailed Student’s *t* test. Significance levels are indicated as ∗*p* < 0.05, ∗∗*p* < 0.01, ∗∗∗*p* < 0.001, ∗∗∗∗*p* < 0.0001.

Because endocrine status may influence aging-related metabolic remodeling, we conducted a panel of assays to assess serum biochemical and reproductive hormone profiles. Specifically, we quantified serum creatine kinase and its MB isoenzyme, and performed liver function tests (albumin, total bile acids, and γ-glutamyl transferase) and renal function tests (urea, uric acid, and creatinine). We found that #11a did not significantly alter these indices (Figures S6A–S6C). Moreover, the fed-state insulin, glucose, and glycated serum protein (GSP) levels in the plasma remained unchanged between aged mice treated with #11a and controls (Figure S6D). In addition, the plasma lipid profile, including triglyceride (TG), cholesterol (CHO), and HDL-cholesterol (HDL-C), remained comparable between vehicle control and #11a-treated mice (Figure S6E). Circulating reproductive hormones, including estradiol, testosterone, follicle-stimulating hormone (FSH), and luteinizing hormone (LH), also showed no statistically significant differences between groups, although estradiol and testosterone both showed a trend toward higher levels in the #11a-treated mice (Figures S6F and S6G). These hormone findings are therefore interpreted as exploratory rather than as evidence for a definitive hormone-mediated mechanism.

### Inhibition of AEP reduces senescence- and inflammation-associated readouts in multiple organs

Senescent cells express high levels of a specific lysosomal β-galactosidase enzyme. Senescence-associated β-galactosidase (SA-β-Gal) is a widely used and classic biomarker for identifying senescent cells in aged tissues and cultures. Accordingly, we performed SA-β-Gal staining in the liver, lung, and iWAT, and representative images suggested reduced senescence-associated staining in #11a-treated tissues (Figure 5A). Masson’s trichrome stain is a histological staining primarily used to distinguish collagen from other tissues, especially muscle and epithelial cells. Its main purpose is to visualize and assess fibrosis in a tissue. Masson staining suggested reduced fibrosis-associated changes in both the liver and kidney, which were further evaluated by the quantified molecular readouts shown below (Figure 5B). Cellular senescence is associated with cell cycle arrest. The tumor suppressors p53 and p16 are key senescence effectors; p53 acts largely through p21, whereas p16 directly inhibits CDK4/6. Immunoblotting analysis showed that these senescent biomarkers showed a decreasing trend in both the liver and kidney after #11a treatment, whereas they displayed variation in the lung (Figures 5C–5E). RT-qPCR analysis further validated that both *p21*^*WAF1*^ and *p16*^*Ink4a*^ mRNA expression levels were significantly decreased in the kidney (Figures 5F and 5G). IF staining further confirmed that p21, p16, and active AEP levels were notably repressed by #11a, whereas Lamin B1 was elevated, in both kidney and liver(Figures 5H–5K). AEP activity is upregulated by inflammatory cytokines and is implicated in the processing of components in inflammatory pathways, creating a vicious cycle where aging-related inflammation promotes AEP activity, which in turn exacerbates inflammatory damage.^34^^,^^35^ Consequently, blockage of AEP with #11a in aged mice substantially decreased IL-1β, IL-6, and TNF-α levels in brain, muscle, liver, lung, and kidney tissues from the aged mice compared to control mice (Figures 5L–5P).

**Figure 5:**
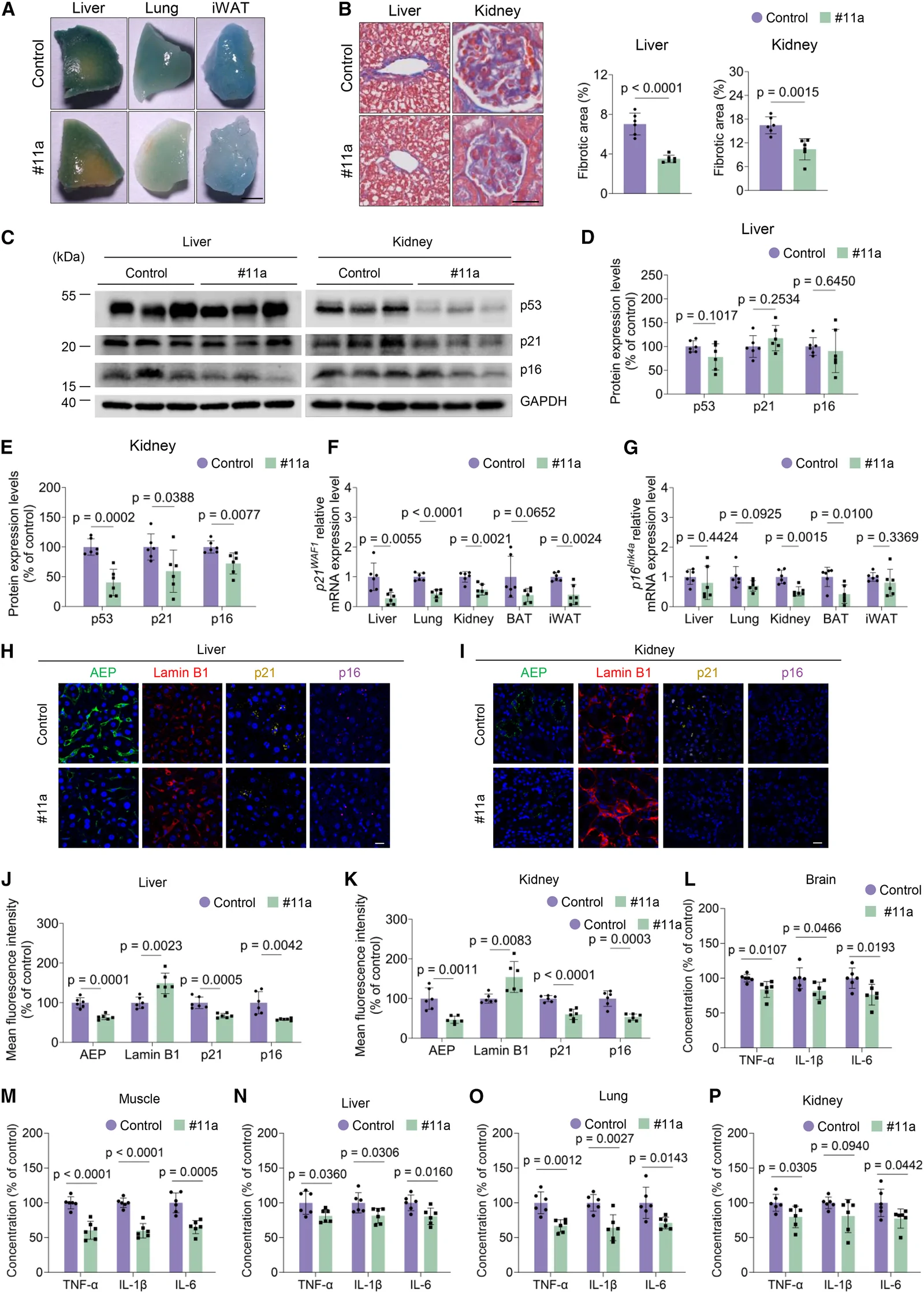
Inhibition of AEP reduces senescence- and inflammation-associated readouts in multiple organs (A) Representative SA-β-Gal staining images of liver, lung, and iWAT from 23-month-old control and #11a-treated mice. (B) Representative Masson’s trichrome staining of liver and kidney tissues, with quantification where indicated (*n* = 6). Scale bars: 50 μm. (C–E) Western blot analysis (C) and quantification (D and E) of p53, p21, and p16 protein levels in the liver (D) and kidney (E) (*n* = 6). (F and G) qPCR analysis of p21 (F) and p16 (G) mRNA expression in the liver, lung, kidney, BAT, and iWAT (*n* = 6). (H–K) IF staining (H and I) and quantification (J and K) of AEP, Lamin B1, p21, and p16 in liver and kidney sections (*n* = 6). Scale bars: 50 μm. (L–P) Concentrations of TNF-α, IL-1β, and IL-6 in brain (L), muscle (M), liver (N), lung (O), and kidney (P). Data are presented as mean ± SD. Statistical significance was determined by unpaired two-tailed Student’s *t* test. Significance levels are indicated as ∗*p* < 0.05, ∗∗*p* < 0.01, ∗∗∗*p* < 0.001, ∗∗∗∗*p* < 0.0001.

### #11a mitigates cellular senescence and reduces NAMPT cleavage by AEP

To further assess the effect of #11a in a stress-associated cellular model, we treated HT22 cells with H_2_O_2_ in the presence or absence of #11a and found that #11a robustly suppressed the H_2_O_2_-induced increase in SA-β-Gal staining (Figure 6A). Immunoblotting analysis revealed that H_2_O_2_ strongly induced AEP activation and increased NAMPT C137 fragmentation, both of which were attenuated by #11a, accompanied by SIRT1 elevation (Figures 6B and 6C). Inflammatory cytokines, including *Il1b*, *Il6*, and *Tnfa*, were also substantially elevated by H_2_O_2_ and were reduced by #11a (Figure 6D). Together, these data indicate that #11a attenuates several stress- and senescence-associated readouts and diminishes NAMPT cleavage in this model. Because cells were collected 24 h after H_2_O_2_ exposure, these data should be interpreted as reflecting an early stress- and senescence-associated response rather than a fully established senescence program. In this acute setting, the NAD^+^/NADH ratio remained largely unaltered, yet #11a was still able to limit NAMPT cleavage and blunt the early senescence-like changes (Figure 6E).

**Figure 6:**
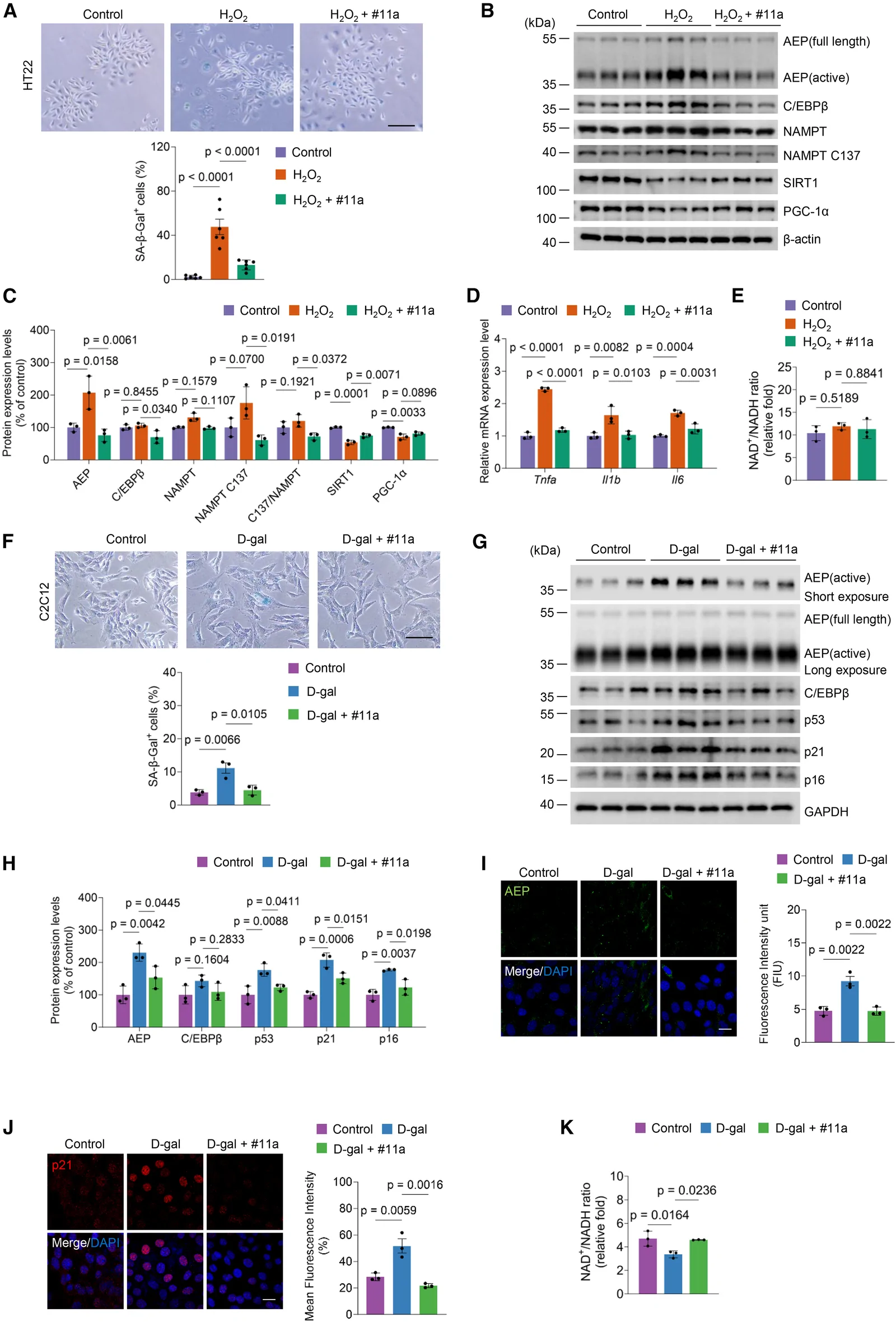
#11a attenuates senescence-associated and stress-related phenotypes *in vitro* and reduces NAMPT cleavage (A) Representative images and quantification of SA-β-Gal staining of HT22 cells in the indicated groups. DMSO was used as a vehicle control (*n* = 3). Scale bars: 100 μm. (B and C) Western blot (B) and quantification (C) of AEP, C/EBPβ, NAMPT C137, SIRT1, and PGC-1α protein expression in the H_2_O_2_-induced stress-associated model of HT22 cells (*n* = 3). (D) qPCR analysis of *Tnfa*, *Il1b*, and *Il6* mRNA levels in the HT22 cells (*n* = 3). (E) NAD^+^/NADH ratio was also measured in HT22 cells exposed to H_2_O_2_ with or without #11a (*n* = 3). (F) Representative images and quantification of SA-β-Gal staining of C2C12 cells in the indicated groups. DMSO was used as a vehicle control (*n* = 3). Scale bars: 100 μm. (G and H) Western blot (G) and quantification (H) of AEP, C/EBPβ, p53, p21, and p16 protein levels in a D-galactose (20 mg/mL)-induced senescence model of C2C12 cells (*n* = 3). (I and J) IF staining of AEP (I) and p21 (J) in C2C12 cells (*n* = 3). Scale bars: 100 μm. (K) NAD^+^/NADH ratio was also measured in C2C12 cells exposed to D-galactose with or without #11a (*n* = 3). Data are presented as mean ± SD. Statistical significance was determined by ordinary one-way ANOVA with Bonferroni’s multiple-comparison test. Significance levels are indicated as ∗*p* < 0.05, ∗∗*p* < 0.01, ∗∗∗*p* < 0.001, ∗∗∗∗*p* < 0.0001.

D-galactose (D-gal) is commonly used experimentally to induce accelerated aging-like and senescence-like phenotypes through oxidative stress. We next assessed #11a in C2C12 cells, an immortalized murine myoblast cell line, exposed to D-gal. Again, #11a significantly repressed the D-gal-induced increase in SA-β-Gal staining (Figure 6F). Immunoblotting showed that D-gal conspicuously escalated AEP activation in C2C12 cells. Furthermore, p53, p21, and p16 were all correspondingly elevated, and these effects were greatly blunted by #11a (Figures 6G and 6H). The biochemical events of AEP activation and p21 elevation after D-gal treatment were further substantiated by IF staining, and the fluorescent intensities of active AEP and p21 were dramatically attenuated by #11a (Figures 6I and 6J). In addition, D-galactose reduced the NAD^+^/NADH ratio in C2C12 cells, and #11a restored this ratio, supporting a context-dependent effect on cellular NAD^+^ redox balance (Figure 6K). Hence, #11a attenuates several senescence-associated features and reduces active AEP-associated NAMPT cleavage in this cellular model.

## Discussion

Our findings support the idea that AEP is involved in multiple aging-associated pathological processes and that pharmacological inhibition of AEP may ameliorate several age-related phenotypes. In the current work, #11a alleviated multiple frailty indices in both male and female mice and was associated with attenuated age-related decline in physical and cognitive functions. In parallel, #11a reduced NAMPT cleavage and was associated with higher NAD-related readouts together with elevated SIRT1 and PGC-1α levels. #11a treatment was also associated with improved insulin sensitivity and reduced adiposity-related changes in BAT and iWAT. Furthermore, #11a reduced SA-β-Gal staining and lowered several senescence- and inflammation-associated markers in multiple organs. Together, these observations are consistent with our recent report that administration of nicotinamide mononucleotide (NMN) or #11a ameliorates age-related disorders and extends lifespan in Thy1-C/EBPβ transgenic mice and worms, further supporting AEP as a therapeutically relevant target.^28^

NAD^+^ is a critical enzymatic co-factor implicated in numerous biological pathways. NAMPT acts as the rate-limiting enzyme in the biosynthesis of NAD^+^ from nicotinamide.^27^ Within forebrain excitatory neurons, intracellular NAMPT-mediated biosynthesis is the principal mechanism for maintaining NAD^+^ levels, which are crucial for neuronal survival and function.^36^ Mice with a conditional knockout of NAMPT in the forebrain excitatory neurons (CaMKII*α*-NAMPT^−/−^) develop hippocampal and cortical atrophy, astrogliosis, microgliosis, and aberrant CA1 dendritic morphology, concomitant with significant motor and cognitive deficits.^36^ The critical role of NAMPT is further highlighted by findings that its knockdown in the CA1 region replicates age-associated cognitive phenotypes, which are subsequently ameliorated by NMN administration.^36^ Therapeutic augmentation of NAD^+^ levels utilizing precursors such as nicotinamide riboside (NR) and NMN mitigates cognitive deficits, Aβ pathology, and Tau pathology across various AD animal models and in AD-induced pluripotent stem cell (iPSC)-derived neurons.^32^^,^^37^^,^^38^ Furthermore, NAD^+^ augmentation restores mitochondrial function and ameliorates age-related decline.^32^ The functional significance of NAD^+^ is largely mediated through sirtuins, an evolutionarily conserved family of NAD^+^-dependent deacetylases that are integral to the regulation of aging and longevity.^29^^,^^30^ Accordingly, our data support a model in which inhibition of AEP via #11a suppresses NAMPT cleavage and is associated with improved NAD-related readouts, thereby potentially contributing to reduced senescence-associated changes (Figures 3, 6, and S4). Although these findings are consistent with a model in which #11a modulates the AEP-NAMPT-NAD^+^/SIRT1/PGC-1α pathway, the current evidence remains largely correlative in naturally aged mice. Additional causal studies will be needed to determine the extent to which this pathway is required for the survival and health span phenotypes observed here. To further examine whether the NAD-related effects of #11a might be explained by altered activity of other major NAD-consuming pathways, we assessed CD38 NADase activity and global PAR levels as functional pathway readouts. Under the tested conditions, #11a did not significantly alter CD38 NADase activity or global PARylation in brain and quadriceps tissues (Figure S5). These observations do not establish direct target engagement of CD38 or PARP by #11a.

CCAAT/enhancer binding proteins (C/EBPs) belong to the basic-leucine zipper DNA-binding protein family and are implicated in central nervous system (CNS) inflammation.^39^ Accumulating evidence positions C/EBPβ as a principal regulator of the aging process. It plays a critical role in mediating oncogene-induced senescence and the SASP, particularly the expression of pro-inflammatory cytokines and chemokines.^40^ C/EBPβ also cooperates with NF-κB to activate SASP-related genes in senescent cells.^41^ Analyses of senescence-related genes across human tissues consistently identify the cell cycle regulator *CDKN1A (p21)* and SASP regulators such as *CEBPB* and *MAP2K3*.^42^^,^^43^^,^^44^ Bioinformatic studies further associate p53, BDNF, CDKN1a, and C/EBPβ with brain aging in senescence-accelerated mouse P8 (SAMP8) models,^45^ while statistical evidence substantiates the influence of *cebpb* on murine aging.^46^ Additional findings implicate C/EBPβ in health span and lifespan determination. For instance, age-related DNA methylation changes are linked to aging, and impaired demethylation at C/EBPβ-binding sites can induce premature aging phenotypes.^47^ Notably, *Cebpb* is a top candidate in screens for transcriptional regulators of aging-associated genes.^48^ C/EBPβ is upregulated in AD brains.^49^ We have shown that it temporally regulates AEP expression, which cleaves both APP N585 and Tau N368, triggering AD pathogenesis.^10^^,^^26^^,^^35^^,^^50^

Recently, our work has demonstrated that neuronal-specific overexpression of C/EBPβ in Thy1-C/EBPβ transgenic mice recapitulates age-associated phenotypes and reduces lifespan.^51^ Most recently, we showed that the age-associated upregulation of neuronal C/EBPβ is a determinant of frailty and lifespan, acting through the inactivation of NAMPT by AEP proteolytic cleavage at N136. Elevated C/EBPβ expression promotes neuronal senescence and loss, a process mechanistically linked to the proteolytic cleavage of NAMPT by active AEP, culminating in the depletion of NAD^+^. Genetic ablation of AEP or expression of an AEP-resistant NAMPT N136A mutant significantly extends the lifespan of neuronal-specific Thy1-C/EBPβ transgenic mice.^28^ Concordantly, neuronal overexpression of the *C. elegans* C/EBPβ ortholog, *cebp-2*, reduces lifespan and diminishes NAD^+^ levels, effects that are rescued by administration of NMN or the AEP inhibitor #11a. Treatment with either NMN or #11a substantially ameliorates cognitive and motor deficits in Thy1-C/EBPβ mice and enhances their life expectancy. Notably, inhibitor #11a demonstrates superior efficacy to NMN in mitigating the accelerated aging phenotype observed in this transgenic model.^28^ AEP cleaves the PP2A inhibitor SET.^12^ This cleavage hyperactivates PP2A, leading to the dephosphorylation and destabilization of the transcription factor EB (TFEB). This disrupts lysosomal biogenesis and autophagic flux, two processes essential for cellular rejuvenation and the clearance of damaged proteins and organelles.^52^ By cleaving SET and disrupting TFEB function, AEP directly impairs autophagy and lysosomal functions. This leads to the accumulation of cellular debris, dysfunctional mitochondria, and aggregated proteins, a hallmark of aged cells. Consequently, therapeutic targeting of AEP may represent a novel and promising strategy for counteracting aging and its associated pathologies.

Kidney aging is associated with molecular and physiological changes that cause a loss of renal function and of regenerative potential.^53^ AEP is highly expressed in the renal tubules, and it is significantly downregulated with the growth of age. Notably, tubule-specific AEP-knockout mice exhibit spontaneous renal interstitial fibrosis from the 3rd month. In the tubule-specific AEP-knockout mice and the cultured AEP-knockdown HK-2 cells, AEP deficiency induces the activation of tubular senescence and thus increases the secretion of profibrotic senescence-associated cytokines, which in turn accelerates the activation of fibroblasts. Blockage of senescence mitigates the fibrotic lesion caused by AEP deficiency. Remarkably, silencing AEP leads to the elevated lysosome pH value, enlargement of lysosome size, and an increase of lysosomal voltage-dependent membrane channel proteins. AEP downregulation induces nuclear TFEB activation, accompanied by impaired mitophagy and increased mitochondrial reactive oxygen species (mtROS) accumulation.^54^ In alignment with these observations, AEP protects against podocyte injury through modulating the dynamics of the cytoskeleton via cleaving cofilin-1. AEP is highly upregulated in diabetic glomeruli and hyperglycemic stimuli-treated podocytes; however, AEP gene knockout and its inhibitor treatment accelerate diabetic nephropathy (DN) in streptozotocin-induced diabetic mice, whereas specific induction of AEP in glomerular cells attenuates podocyte injury and renal function deterioration.^55^^,^^56^ These observations highlight that the physiological roles of AEP are tissue- and disease-context dependent, and they underscore the need for careful assessment of tolerability when AEP is inhibited chronically. In the present study, CBC analysis did not reveal major abnormalities in the assessed systemic indices within the evaluated time window (Table S1), although these datasets do not establish long-term safety beyond the current study duration. These results are consistent with previous reports that partial reduction of AEP activity can be tolerated without overt phenotypes.^57^ Therapeutic NAD^+^ augmentation also exhibits restorative effects on mitochondrial function and age-related decline.^32^

An intriguing observation in our aging cohort was that female mice exhibited a shorter lifespan compared with males in both the control and #11a-treated groups, although the inhibitor significantly extended lifespan in both sexes. This trend contrasts with the commonly reported longer lifespan of female C57BL/6 mice in many studies,^58^^,^^59^ but it is not without precedent. The manifestation of sex differences in lifespan can depend strongly on pathogen burden, housing conditions, and the spectrum of spontaneous pathologies within a colony.^60^^,^^61^^,^^62^ In some aging C57BL/6 colonies, female mice exhibit a higher incidence of fatal conditions, such as ulcerative dermatitis, which can significantly shorten their median lifespan compared with that of males. Furthermore, reproductive history is a critical and often overlooked determinant.^63^ Unlike virgin females, those that have undergone repeated cycles of pregnancy and lactation experience substantial physiological stress and metabolic costs, which can lead to accelerated aging and a shortened lifespan.^64^^,^^65^ The specific pre-study history of our cohort, acquired at 18 months of age, could therefore have contributed to the observed sex difference. The FI data, showing that #11a ameliorated distinct sets of pathologies in each sex (Figures 1E and 1F), further support the notion that the underlying drivers of aging and mortality likely differed between males and females in this cohort.

Mechanistically, these sex-dimorphic responses may reflect differences in hormonal regulation and inflammatory landscapes. Sex hormones, including estrogen, have been implicated in mitochondrial function, NAD+ metabolism, and SIRT1-associated pathways, which are closely related to the NAMPT/NAD+/SIRT1 axis modulated by AEP inhibition.^29^^,^^30^^,^^66^ In parallel, females often exhibit stronger immune and SASP-associated inflammatory responses involving NF-κB and C/EBPβ signaling, which may alter their sensitivity to interventions targeting the C/EBPβ/AEP axis.^40^^,^^41^^,^^58^ Moreover, sex-dependent differences in metabolic stress, mitochondrial homeostasis, and lysosomal function may further influence AEP activation and downstream therapeutic efficacy.^15^^,^^58^^,^^67^ Together, our work shows that inhibition of AEP via #11a is associated with improved metabolic and frailty-related outcomes and with extended lifespan and health span under the conditions tested.

### Limitations of the study

This study has several limitations. First, food intake was measured at the cage level. Although confirmatory analysis of the balanced months 1–5 window did not identify a significant treatment effect, food intake was numerically lower in #11a-treated cages at several time points. The limited number of cages does not exclude a contribution of altered food intake to the observed phenotypes. Moreover, cage-level food-intake records could not be reliably linked to individual longitudinal frailty, survival, GTT, or ITT outcomes; therefore, valid individual-level food-intake-adjusted analyses could not be performed. A prospectively designed pair-feeding study will be required to distinguish direct pharmacological effects of #11a from food-intake-related effects. Second, both male and female mice were used. Unless otherwise indicated, male and female mice were pooled for statistical analyses, and sex was not included as an independent factor in the primary models. The study was not powered to detect sex-specific effects for all endpoints. Although sex-specific frailty data are presented in Figures 1E and 1F, potential sex-dependent effects of #11a on other outcomes cannot be excluded. Future studies with adequate power should evaluate sex as a biological variable. Third, HT22 cells were obtained without supplier annotation of sex; therefore, sex-related effects in this cell model could not be assessed. C2C12 cells are of female origin. Fourth, although our molecular findings are consistent with modulation of the AEP-NAMPT-NAD^+^/SIRT1 pathway, the mechanistic evidence in naturally aged mice remains largely correlative and does not establish pathway necessity. We included functional readouts of CD38 NADase activity and PARylation as alternative NAD^+^-consuming pathways; however, future studies using NAMPT genetic manipulation and broader NAD metabolomics (e.g., NMN, NR, nicotinamide) will be required to formally establish pathway necessity. Fifth, the *in vitro* models capture stress- and senescence-associated responses but do not fully define a complete senescence program under all conditions tested. Finally, several tissue-level analyses and tolerability assessments remain limited in scope, and broader quantitative pathology together with more comprehensive long-term safety evaluations will be needed in future studies.

## Resource availability

### Lead contact

Requests for further information and resources and reagents should be directed to and will be fulfilled by the lead contact, Keqiang Ye (yekeqiang@suat-sz.edu.cn).

### Materials availability

The AEP inhibitor #11a used in this study is available upon request from the lead contact.

### Data and code availability

• All data reported in this paper will be shared by the lead contact upon request.
• No original code has been generated in this study.
• Any additional information required to reanalyze the data reported in this paper is available from the lead contact upon request.

## Acknowledgments

This work was supported by the 10.13039/501100001809National Natural Science Foundation of China (82394431), the 10.13039/501100012166National Key R&D Program of China (2023YFC3605400), the Shenzhen Science and Technology Program (KQTD20221101093608028), and the Shenzhen SMART Medical Research Project (B2404001) to K.Y.

## Author contributions

K.Y. conceived and supervised this study. M.Y., X.M., Z.X., M.W., and Z.Q. were responsible for all aspects of study design. M.Y. and X.M. conducted the experiments, interpreted the results, and performed the statistical analysis. K.Y., M.Y., and X.M. wrote the manuscript. M.Y., X.M., Z.X., M.W., and Z.Q. verified the underlying data. All authors critically reviewed the manuscript and approved the final version.

## Declaration of interests

K.Y. is an inventor on patent applications related to AEP inhibitors.

## STAR★Methods

### Key resources table

**Table:** 

REAGENT or RESOURCE	SOURCE	IDENTIFIER
**Antibodies**		

Legumain (D6S4H) Rabbit Monoclonal Antibody	Cell Signaling Technology	Cat# 93627; RRID: AB_2800205
Mouse Legumain/Asparaginyl Endopeptidase Antibody	R&D	Cat# AF2058; RRID: AB_2234536
Alpha SMA Antibody	Affinity Biosciences	Cat# BF9212; RRID: AB_2839428
APP N	MERCK	Cat# MAB348; RRID:AB_2056583
APP N585	Ye LAB	N/A
Tau 5	Thermo Fisher	Cat# AHB0042; RRID:AB_2536235
Tau N368	Ye LAB	N/A; N/A
Recombinant Anti-GAPDH antibody (Rabbit mAb)	Servicebio	Cat# GB15004; RRID:AB_2943040
beta Actin Monoclonal Antibody	Thermo Fisher Scientific	Cat# MA1-140; RRID:AB_2536844
C/EBP beta (LAP) Antibody	Cell Signaling Technology	Cat# 3087; RRID: AB_2078052
C/EBP beta Antibody (H-7)	Santa Cruz	Cat# sc-7962; RRID: AB_626772
NAMPT/PBEF Monoclonal antibody	Proteintech	Cat# 66385-1-Ig; RRID: AB_2881761
NAMPT C137	Transgene	N/A
SirT1 (1F3) Mouse Monoclonal Antibody	Cell Signaling Technology	Cat# 8469; RRID:AB_10999470
PGC-1 alpha (3G6) Rabbit Monoclonal Antibody	Cell Signaling Technology	Cat# 2178; RRID:AB_823600
p21 Cip1 Antibody	Affinity Biosciences	Cat# AF6290; RRID:AB_2827699
Purified Mouse Anti-p21	BD	Cat# 556431; RRID:AB_396415
Anti-CDKN2A/p16INK4a	Abcam	Cat# ab189034; RRID:AB_2737282
p16INK4a Monoclonal Antibody (1D7D2)	Invitrogen	Cat# MA5-17054; RRID:AB_2538526
p53 (1C12) Mouse Monoclonal Antibody	Cell Signaling Technology	Cat# 2524; RRID:AB_331743
CD38 (E9F5A) Rabbit Monoclonal Antibody	Cell Signaling Technology	Cat# 68336; RRID:AB_3674060
PARP (46D11) Rabbit Monoclonal Antibody	Cell Signaling Technology	Cat# 9532; RRID:AB_659884
Lamin B1 Antibody (B-10)	Santa Cruz	Cat# sc-374015; RRID:AB_10947408
Anti-PAR (Ab-1) Mouse mAb	Sigma Aldrich	Cat# AM80; RRID:AB_2155072

**Chemicals, peptides, and recombinant proteins**		

#11a	Ye LAB	N/A
H_2_O_2_	Shanghai Lingfeng Chemical Reagent	7722–84–1
D-gal	MCE	HY-N0210
TRIzol^TM^ Reagent	Invitrogen	Cat#15596018CN
DMSO	Thermo Fisher	85190
Z-Ala-Ala-Asn-AMC	Bachem	149697–16–5
CD38 inhibitor 1 (78c)	MCE	HY-123999
Olaparib	MCE	HY-10162
1,N^6^-ethenoadenine dinucleotide (ε-NAD)	MCE	HY-137592
Recombinant Human CD38 Protein	SinoBiological	10818-H08H

**Critical commercial assays**		

EnzyFluo™ NAD/NADH Assay Kit	BioAssay Systems	EFND-100
Senescence β-galactosidase staining kit	Cell Signaling Technology	#9860
Pierce™ BCA Protein Assay Kits	Thermo Fisher	23225
Mouse TNF-α ELISA Kit	Elabscience	E-EL-M3063
Mouse IL-1β ELISA Kit	Elabscience	E-EL-M0037
Mouse IL-6 ELISA Kit	Elabscience	E-EL-M0044

**Experimental models: Cell lines**		

HT22	CCTCC	SCSP-5419
C2C12	CCTCC	SCSP-505

**Experimental models: Organisms/strains**		

C57BL/6	Aniphe Biolaboratory Inc	N/A

**Oligonucleotides**		

*Adrb3*-Forward	Sangon	CTTGCCTCTCCATTCCTGACATC
*Adrb3*-Reverse	Sangon	ATTCCGCACGACACACTTCACC
*Adipoq*-Forward	Sangon	TCTGCCTCAGTTTCTCTCGGCT
*Adipoq*-Reverse	Sangon	GTAATAGAGCCAGGGAACGAAGC
*Leptin*-Forward	Sangon	GCAGTGCCTATCCAGAAGTCC
*Leptin*-Reverse	Sangon	GGAATGAAGTCCAAGCCAGTGAC
*Glut4*-Forward	Sangon	TTCCTGTGGAGCCTTGTCACCA
*Glut4*-Reverse	Sangon	CTCCACCTTCTGTCTCTGCTCA
*Insr*-Forward	Sangon	AGATGAGGTGCAGTGTGGCT
*Insr*-Reverse	Sangon	GGTTCCTTTGGCTCTTGCCACA
*Fh1*-Forward	Sangon	GAACTCACACGCAGGATGCTGT
*Fh1*-Reverse	Sangon	GGCGGCTTTTATTCTCACCATCG
*Pparg*-Forward	Sangon	GTACTGTCGGTTTCAGAAGTGCC
*Pparg*-Reverse	Sangon	ATCTCCGCCAACAGCTTCTCCT
*Pgc1α*-Forward	Sangon	GAATCAAGCCACTACAGACACCG
*Pgc1α*-Reverse	Sangon	CATCCCTCTTGAGCCTTTCGTG
*Ucp1*-Forward	Sangon	GCTTTGCCTCACTCAGGATTGG
*Ucp1*-Reverse	Sangon	CCAATGAACACTGCCACACCTC
*Dio2*-Forward	Sangon	GGTGGTCAACTTTGGTTCAGCC
*Dio2*-Reverse	Sangon	AAGTCAGCCACCGAGGAGAACT
*p21*-Forward	Sangon	TCGCTGTCTTGCACTCTGGTGT
*p21*-Reverse	Sangon	CCAATCTGCGCTTGGAGTGATAG
*p16*-Forward	Sangon	TGTTGAGGCTAGAGAGGATCTTG
*p16*-Reverse	Sangon	CGAATCTGCACCGTAGTTGAGC
*Tnfα*-Forward	Sangon	GGTGCCTATGTCTCAGCCTCTT
*Tnfα*-Reverse	Sangon	GCCATAGAACTGATGAGAGGGAG
*Il1b*-Forward	Sangon	TGGACCTTCCAGGATGAGGACA
*Il1b*-Reverse	Sangon	GTTCATCTCGGAGCCTGTAGTG
*Il6*-Forward	Sangon	TACCACTTCACAAGTCGGAGGC
*Il6*-Reverse	Sangon	CTGCAAGTGCATCATCGTTGTTC
*Gapdh*-Forward	Sangon	CATCACTGCCACCCAGAAGACTG
*Gapdh*-Reverse	Sangon	ATGCCAGTGAGCTTCCCGTTCAG

**Software and algorithms**		

Prism 10	Graphpad	https://www.graphpad.com/
EthoVision XT 17	Noldus	N/A

### Experimental model and study participant details

#### Mice

Wild-type C57BL/6 mice were obtained from Aniphe Biolaboratory Inc (Nanjing, China). Mice began the study at approximately 18 months of age; intermediate phenotyping and tissue collection were performed at 23 months of age. All mice were housed on a 12 h light/dark cycle and kept at 20∼22°C. All animal procedures were approved by the Institutional Animal Care and Use Committee (IACUC) of Shenzhen Institutes of Advanced Technology (SIAT), Chinese Academy of Sciences (approval number: SIAT-IACUC-20220407-SLG(C)-SMJKXY-YKQ-A2096-01), and all experiments conformed to AAALAC International guidelines and relevant national and institutional regulatory standards. Both male and female C57BL/6 mice were used. Unless otherwise indicated, male and female mice were pooled for statistical analyses. Sex was not included as an independent factor in the primary analyses, and the study was not powered to detect sex-specific effects for all endpoints. Sex-specific frailty data are presented in Figures 1E and 1F, and sex-associated differences are described in the Results and Discussion. The limited power to evaluate sex as an independent variable is acknowledged as a limitation.

#### Cell lines

HT22 mouse hippocampal neuronal cells (sex not specified by the supplier) and C2C12 mouse myoblasts (female) were cultured in Dulbecco's modified Eagle's medium (DMEM) (Sigma-Aldrich, D7777) supplemented with 10% fetal bovine serum (FBS) (Gibco, 10270106) and 1% penicillin-streptomycin (Gibco, 15140122) at 37°C in a humidified atmosphere of 5% CO_2_. For senescence induction, HT22 cells were treated with 100 μM H_2_O_2_ for 2 h. Following the removal of the medium and a phosphate-buffered saline (PBS) wash, the cells were incubated with 500 nM #11a in DMEM for 24 h, thereby assessing the effects of #11a on cellular aging. C2C12 cells were treated with 20 mg/ml D-galactose for 3 days. #11a was diluted in DMSO supplemented with D-galactose. 500 nM #11a was added to aged C2C12 cells for 24 h, thereby assessing the effects of #11a on cellular aging. For anti-PAR immunoblot validation, HT22 cells were treated with 500 μM H_2_O_2_ alone (as a positive control) or pretreated with 10 μM olaparib (MCE, HY-10162) for 30 min followed by 500 μM H_2_O_2_ for 30 min, to confirm detection of PAR smear signals under the immunoblotting conditions. The cell lines used in this study were obtained from the China Center for Type Culture Collection (CCTCC, Wuhan, China) and were used within a limited range of low passages after receipt. Neither line is listed in the ICLAC register of commonly misidentified cell lines. The cell lines were not independently authenticated in our laboratory. Mycoplasma contamination was not tested.

### Method details

#### Experimental groups and #11a administration

All lifespan and health span experiments were started at approximately 18 months of age. The naturally aged cohort included both female and male C57BL/6 mice. Health span and survival analyses were initiated concurrently. #11a was administered continuously via the chow diet at 0.056 g/kg diet. Based on an average food intake of ∼4 g/day for a ∼30 g mouse, the estimated daily exposure was ∼7.5 mg/kg body weight/day. Mice were randomly assigned to control (standard diet, LAD-3001M) or #11a-supplemented diet groups (*n* = 26 per group). All mice were assigned at approximately 18 months of age to either the control diet or the #11a-supplemented diet group. A pre-specified subset of animals from each group was euthanized after five months of treatment (23 months of age) for intermediate phenotyping and tissue collection. The remaining animals continued under the same treatment conditions for longitudinal lifespan and frailty assessment. Animals removed for planned intermediate tissue collection were not included as spontaneous deaths in survival analyses.

#### Randomization and blinding

Mice were randomly assigned to control or #11a treatment groups at the beginning of the study. Lifespan monitoring was conducted continuously throughout the study period. Frailty index scoring, behavioral assessments, histological analyses, and image quantifications were performed by investigators blinded to treatment allocation whenever feasible. Data analysis was conducted using coded samples prior to group unblinding.

#### Lifespan and frailty assessment

##### Lifespan analysis

Mouse lifespan was monitored as previously described. The primary outcome of the lifespan study was overall survival with age at death recorded for mice that died spontaneously or were humanely euthanized after reaching predefined endpoint criteria. Euthanasia was performed only when mice met IACUC-approved humane endpoint criteria, based on an independent assessment by a veterinarian in accordance with AAALAC International guidelines, and were considered unlikely to survive beyond the next 48 hours, without significant discomfort. Humane endpoint criteria included severe lethargy, rapid weight loss (over two weeks > 20%), severe distended abdomen, body condition score with signs of pain (e.g., grimacing), unresponsiveness to external stimuli, severe ulcerated or bleeding tumors, and pronounced hypothermia with abnormal respiratory rate. Body weight was recorded twice monthly. Food intake was assessed monthly at the cage level; three repeated measurements per cage were averaged to generate one cage-level value at each time point.

##### Frailty assessment

Frailty was assessed using a validated non-invasive clinical frailty index, as previously described.^68^ For the subjective properties of the assessments, all measurements were completely blinded. These assessments indicate age-associated deterioration of health and include evaluation of the animal musculoskeletal system, the vestibulocochlear/auditory systems, the ocular and nasal systems, the digestive system, the urogenital system, the respiratory system, signs of discomfort and body weight. A score of 0 indicates no frailty signs and full health for a given trait; moderate and severe phenotypes are scored as 0.5 and 1, respectively. To assess the effect of #11a on health span, frailty index scores were recorded monthly starting at 18 months of age and compared between #11a-fed and control groups throughout their lifespans.

#### Assessment of neuromuscular function and motor coordination

##### Treadmill test

For the exhaustive tests, mice were subjected to a graded treadmill running protocol. The test began at a speed of 5 m/min for 3 minutes, followed by incremental increases in velocity at a rate of 2 m/min until reaching 15 m/min. Running time and velocity were recorded for each mouse until exhaustion, defined as the inability to maintain running and subsequently falling off the treadmill. For the endurance tests, mice were run at a constant speed of 15 m/min. Running duration was recorded similarly, as described above, until the mice reached failure.

##### Wire hanging test

Forelimb grip strength and neuromuscular function were assessed using a wire hanging test. Each mouse was gently lifted by the base of the tail and allowed to grasp a stainless-steel grid with both forepaws and hind paws. The grid was then inverted, and the latency to fall was recorded. Each mouse underwent three consecutive trials, and the maximum hanging duration achieved among the trials was used for analysis.

#### Assessment of cognitive function

##### Open field test

Locomotor activity and anxiety-like behavior were assessed using an open field test. The open field box consisted of a square white box (40 cm x 40 cm x 40 cm). Each mouse was placed individually into the center of the arena and allowed to explore freely for 5 minutes. Total distance moved and time spent in the center zone versus periphery were recorded and analyzed using video tracking software (EthoVision, Noldus Information Technology).

##### Novel object recognition test

The novel object recognition test was conducted to evaluate recognition memory. During the familiarization phase, mice were placed in the testing arena for 5 minutes with two identical objects positioned in opposite quadrants. After a 1 h intertrial interval, one object was replaced with a novel object, and mice were allowed to explore both objects freely for another 5 minutes. Exploration was defined as direct sniffing or touching the object. Behavior was recorded using video tracking software (EthoVision, Noldus Information Technology). Discrimination index was calculated as the time spent exploring the novel object divided by the total exploration time of both objects.

##### Y maze

Spatial working memory was assessed using a Y maze with three identical arms. During the training phase, each mouse was placed into the start arm facing the wall and allowed access to only two arms for 10 minutes, while the third (novel) arm was blocked. The maze was cleaned between each mouse to remove odor cues. After training, the mouse was returned to its home cage. After 1 h, the mouse was returned to the start arm and allowed to explore all three arms for 5 min. The number of entries and time spent in each arm were quantified using the tracking software (EthoVision XT, Noldus Information Technology).

##### Intraperitoneal glucose and insulin tolerance tests (IPGTT and IPITT)

Glucose homeostasis and insulin sensitivity were assessed via intraperitoneal glucose tolerance test (IPGTT) and intraperitoneal insulin tolerance test (IPITT), respectively. Blood glucose levels were measured at indicative timepoints after injection using a blood glucometer (ROCHE ACCU-CHEK^TM^). For the IPGTT, mice were fasted for 12 hours and subsequently injected intraperitoneally with a sterile glucose solution at a dose of 2 g/kg body weight. For the IPITT, mice were fasted for 6 hours and subsequently injected intraperitoneally with human insulin at 0.5 U/kg body weight. Blood glucose levels were measured from the tail vein immediately prior to injection (0 min) and at specified time points thereafter using a Roche Accu-Chek glucometer.

##### Sample collection

After five months of treatment, mice were humanely euthanized via cardiac puncture under deep anesthesia induced and maintained with isoflurane (5% for induction and 2% for maintenance). Whole blood was collected into EDTA-coated tubes and centrifuged at 2,000 × g for 10 minutes at 4°C to isolate plasma. Perfusion was performed using a peristaltic pump system with minor modifications. Approximately 20-30 mL of PBS was perfused at a flow rate of 5 mL/min until the effluent from the right atrium became clear and the liver became pale, indicating complete clearance of blood. Major organs, including the brain, liver, and quadriceps muscle, were promptly dissected. Tissues intended for molecular analysis were flash-frozen in liquid nitrogen and stored at -80°C for subsequent analysis. Samples designated for histological examination were immersion-fixed in 4% paraformaldehyde (PFA) for 48 hours at 4°C and subsequently transferred to 75% ethanol for long-term storage.

##### LC-MS/MS quantification

Plasma concentrations of #11a were quantified using liquid chromatography-tandem mass spectrometry (LC-MS/MS; SHIMADZU 8050). Samples were prepared by protein precipitation with methanol. Calibration standards were freshly prepared for each analytical batch. A linear calibration curve was established over the concentration range of 0.1-500 ng/mL based on the peak area ratio of the analyte to the internal standard.

#### Biochemical and molecular analyses

##### Western blotting analysis

Cells and tissues were washed with ice-cold PBS and lysed on ice for 30 min using WB lysis buffer (Yeasen, 20118ES60) and supplemented with protease and phosphatase inhibitors. Lysates were centrifuged at 15,000 × g for 15 minutes at 4°C. Supernatants were collected and mixed with SDS loading buffer containing 5% β-mercaptoethanol and denatured by boiling for 5 minutes. Proteins were separated by SDS-PAGE and subsequently transferred to polyvinylidene difluoride (PVDF) membranes. The membranes were blocked with 5% non-fat milk prepared in Tris-buffered saline with Tween 20 (TBST) for 1 hour at room temperature and then incubated with specific primary antibodies overnight at 4 °C. After incubation, the membranes were washed and probed with horseradish peroxidase (HRP)-conjugated secondary antibodies for 1 hour at room temperature. After thorough washing with TBST, protein bands were visualized using an enhanced chemiluminescence (ECL) detection system.

##### Quantitative PCR analysis

Total mRNA was isolated from cells and tissues using TRIzol reagent (Invitrogen, 15596018CN). cDNA synthesis was performed with 1 μg of total RNA using one-step RT-gDNA digestion SuperMix (Yeasen, 11142ES60). Quantitative PCR amplification was conducted on QuantStudio 3 System (Applied Biosystems, USA) with SYBR Green Master Mix (Yeasen, 11185ES03) according to the manufacturer’s instructions. Relative mRNA expression levels were calculated via the 2^^ΔΔCt^ method with Gapdh as the endogenous reference gene.

##### Enzyme-linked immunosorbent assay (ELISA)

Plasma levels of insulin, FSH, LH, estradiol, and testosterone were determined using corresponding commercial ELISA kits (Cloud-Clone Corp) according to manufacturer’s protocols. Blood samples were collected from fasted mice and centrifuged at 3000 rpm for 15 min to obtain serum. Plasma samples were diluted 1:10 with the provided sample diluent before analysis. The absorbance was measured at 450 nm by microplate reader (BioTek, Synergy H1, USA), and sample concentrations were determined based on a standard curve and expressed in ng/mL.

Levels of TNF-α, IL-1β, and IL-6 were determined using corresponding commercial ELISA kits (Elabscience) according to manufacturer’s protocols. Tissues were collected at same time. Centrifugation was performed at 5,000 rpm for 10 min at 4 °C then the supernatants were harvested. Samples were diluted with the provided sample diluent before analysis. The absorbance was measured at 450 nm by microplate reader (BioTek, Synergy H1, USA), and sample concentrations were determined based on a standard curve.

##### Immunofluorescence

Tissue samples were fixed in 4% PFA for 24–48 hours at 4°C. Following fixation, tissues were processed through a graded ethanol series, embedded in paraffin and sectioned at a thickness of 4 μm for histological analysis. Alternatively, for cryosectioning, tissues were embedded in optimal cutting temperature (OCT) compound, snap-froze and sectioned at 10 μm. Sections were blocked with 5% bovine serum albumin (BSA) for 1 hour and then incubated with primary antibodies overnight at 4 °C. Alexa Fluor-conjugated secondary antibodies were applied for 1 hour at room temperature. Nuclei were counterstained with DAPI. Coverslips were mounted onto glass slides and imaged using a confocal microscope (LSM 980, Zeiss). The fluorescence density was quantified using ImageJ software. The ratio of the average fluorescence intensity of each group was used to draw the histogram.

##### Masson’s trichrome staining

Tissue samples were fixed in Bouin’s solution (Sigma, #HT10132) for 24 hours, embedded in paraffin, and sectioned at a thickness of 4 μm. Sections were subjected to a sequential staining protocol as follows: Weigert’s hematoxylin (Sigma, #HT1079) for 5 minutes, Biebrich scarlet–acid fuchsin (Sigma, #B5393) for 10 minutes, phosphomolybdic acid (Sigma, #P7960) for 10 minutes, and aniline blue (Sigma, #415049) for 5 minutes. Sections were then differentiated in 1% acetic acid. Collagen deposition was quantified as the percentage of blue-stained area using ImageJ software. The percentage of blue area was calculated as number of aniline blue-positive pixels divided by total tissue pixels, analyzed in at least ten random 20× fields per section.

##### Microcomputed tomography (μCT)

Femurs were scanned *ex vivo* using a micro-CT system (NMC-200, NEMO, China) with an isotropic resolution of 12 μm, operating at 60 kV and 120 μA with a 0.5 mm aluminum filter. Data acquisition was performed using Cruiser software. Images were reconstructed using the FDK algorithm (Recon software) and segmented with a calibrated threshold (790.2 grayscale units, equivalent to 360 mg HA/cm^3^). Trabecular bone was analyzed in a 1.68 mm^3^ region starting 0.6 mm proximal to the growth plate; cortical bone was assessed at the mid-diaphysis. Trabecular bone microarchitecture (including bone volume fraction (BV/TV), trabecular thickness (Tb.Th), trabecular separation (Tb.Sp), trabecular number (Tb.N)) and cortical bone morphology (including cortical thickness (Ct.Th), cortical area (Ct.Ar)) were computed in accordance with ASBMR guidelines. 3D renderings and analysis were generated using Avatar software (μCT Ray v4.0, Scanco Medical).

##### Hematological and biochemical analyses

Whole blood was collected via cardiac puncture into EDTA-coated tubes (Servicebio, #QX0005) for complete blood count analysis (CBC) and into heparinized tubes (Servicebio, #QX0015) for serum biochemistry. Hematological parameters were measured within 2 hours of collection using an automated veterinary blood cell analyzer (Mindray BC-5000vet, China) at ServiceBio Co., Ltd. (Wuhan, China). Serum biochemical parameters were analyzed using a Chemray 800 automatic biochemistry analyzer (Rayto, China). Quality control was performed through daily calibration with commercial standards and an inter-assay coefficient of variation maintained below 5%.

##### Senescence-associated β-galactosidase (SA-β-Gal) activity

SA-β-gal staining was detected using the senescence β-galactosidase staining kit (Cell Signaling Technology, #9860) according to the manufacturer’s instructions. SA-β-gal^+^ cells were quantified as a percentage of the total number of cells.

##### AEP activity assay

Tissue homogenates were incubated in 200 μl assay buffer (20 mM citric acid, 60 mM Na_2_HPO_4_, 1 mM EDTA, 0.1% CHAPS, and 1 mM dithiothreitol, pH 6.0) containing 20 μM δ-secretase substrate, Z-Ala-Ala-Asn-AMC (Bachem). The release of fluorescent AMC resulting from substrate cleavage was quantified kinetically by measuring fluorescence at an emission wavelength of 460 nm using a plate reader maintained at 37 °C.

##### CD38 NADase activity assay

CD38 NADase activity was measured as previously described with minor modifications.^69^^,^^70^^,^^71^ Frozen tissue samples (∼20 mg) were homogenized in 300 μL ice-cold sucrose buffer (0.25 M sucrose, 40 mM Tris, pH 7.4), briefly sonicated, and centrifuged at 14,000 × g for 10 min at 4°C. Protein concentrations in the supernatants were determined by BCA assay, and equal amounts of protein were used for each reaction. ε-NAD (50 μM final concentration) was added to initiate the reaction. Fluorescence was measured at 37°C every minute for 1 h (excitation 360 nm; emission 460 nm). NADase activity was calculated from the slope of the linear portion of the fluorescence-time curve after background subtraction and normalized to protein content. Recombinant human CD38 (Sino Biological, Cat. 10818-H08H) and 78c (50 nM) were used as positive and inhibitor controls, respectively.

##### NAD^+^/NADH assay

Levels of NAD^+^ and NADH in brain, quadriceps muscle, liver, and kidney homogenates were measured using the NAD^+^/NADH Assay Kit (EnzyFluo™, EFND-100) according to the manufacturer’s instructions. Samples were mixed in a 96-well plate at room temperature. Total NADH and NAD^+^ were quantified at λ_ex/em_ = 530/585 nm using a microplate reader.

### Quantification and statistical analysis

The statistical analyses were performed with GraphPad Prism 10.1.2 software. All experiments were repeated at least three times independently with blinded quantification. Data are presented as mean ± SEM or mean ± SD as indicated in figure legends, and n represents biological replicates. Unless otherwise indicated, statistical significance was determined using unpaired two-tailed Student’s t tests for two groups, one-way ANOVA followed by Bonferroni’s multiple-comparison test for three or more groups, and Mann-Whitney U tests when normality could not be assumed. Kaplan-Meier survival curves were compared using the log-rank test. Hazard ratios and 95% confidence intervals were estimated by the Mantel-Haenszel method. Longitudinal frailty index data were analyzed using linear mixed-effects models with mouse ID as a random effect and treatment and time as fixed effects; model fit was compared with that of a simple ANOVA model using a chi-square test. Food-intake data from months 1-5 after treatment were analyzed using a two-way repeated-measures ANOVA, with treatment as the between-cage factor, time as the within-cage factor, and cage as the repeated experimental unit. Greenhouse-Geisser correction and Sidak-adjusted post hoc comparisons were applied. Months 6-9 were not included in the confirmatory analysis because cage availability declined after planned intermediate tissue collection and subsequent natural attrition. Cell-based experiments were repeated at least three times independently. Investigators were blinded to treatment allocation for frailty scoring, behavioral assessments, histological analyses, and image quantification whenever feasible. Significance was defined as *p* < 0.05. Asterisks indicate ∗*p* < 0.05, ∗∗*p* < 0.01, ∗∗∗*p* < 0.001, and ∗∗∗∗*p* < 0.0001.
