# Supplementary material for: Inhibition of asparagine endopeptidase extends the lifespan and improves health span during aging
Source: iScience. 2026 Oct 23;29(11):117990. doi: 10.1016/j.isci.2026.117990 (PMC13720683; doi:10.1016/j.isci.2026.117990)

iScience, Volume *29*

Supplemental information

Inhibition of asparagine endopeptidase extends the lifespan and improves health span during aging

Menghan Yang, Xin Meng, Zhongyun Xie, Mengmeng Wang, Zhengjiang Qian, and Keqiang Ye

Supplemental Information

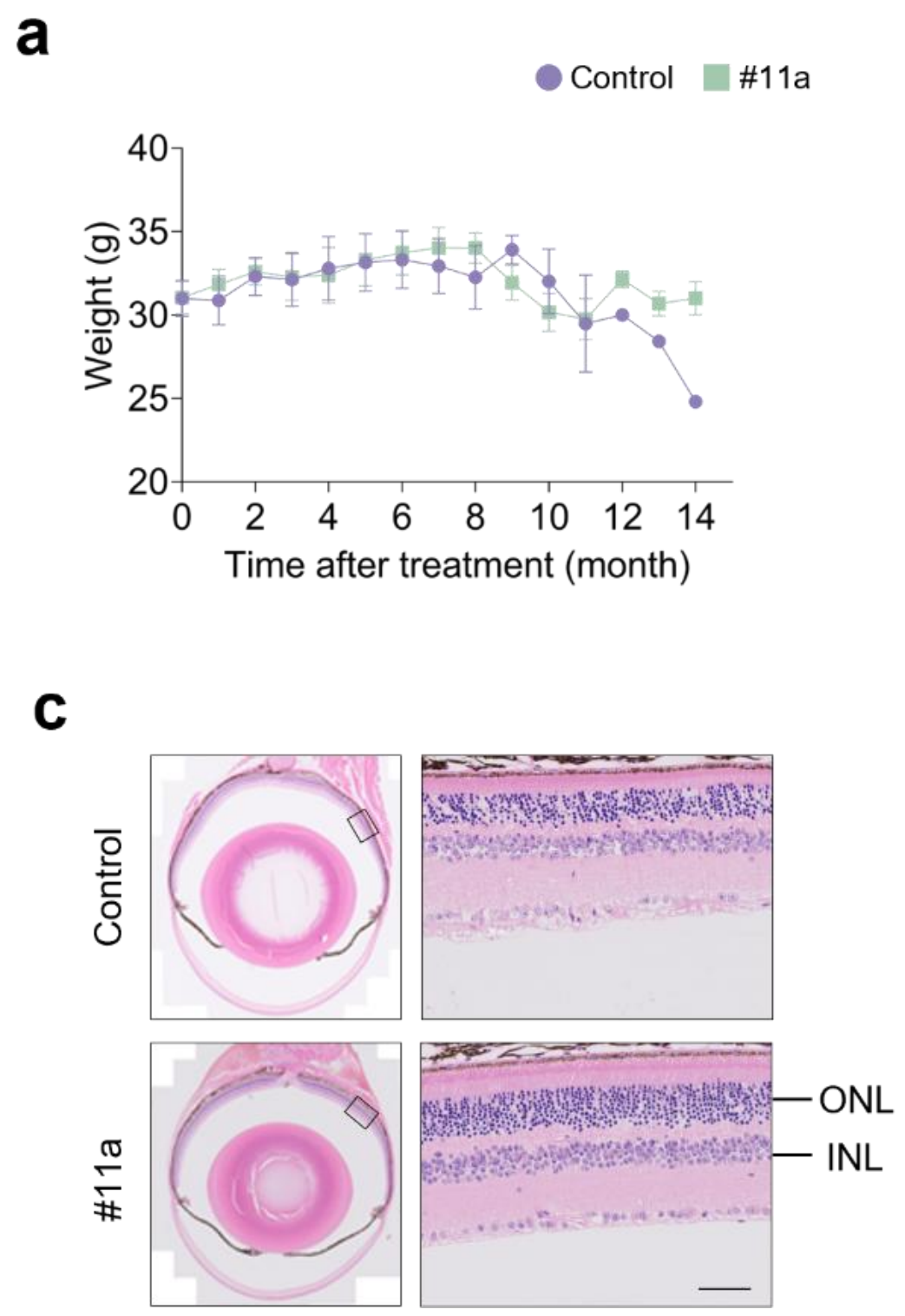

Figure S1. Body weight, food intake, and ocular histology in aged mice.

(a) Body weight trajectories over time. Initial group sizes were control: n = 26, #11a: n = 26; natural attrition occurred during aging. (b) Average daily food intake at the cage level during months 1-5 after treatment. Three measurements per cage were averaged at each time point. Data are mean ± SEM; n = 6 cages per group. Food intake was analyzed by two-way repeated-measures ANOVA with Greenhouse-Geisser correction and Sidak-adjusted post hoc comparisons. (c) Left: Representative H&E images of eye sections from the control group and #11a group. Right: Higher-magnification view of the boxed retinal region, showing retinal cellular architecture. Scale bar: 50 μm. Data are presented as mean ± SEM. Significance levels are indicated as follows: $*p < 0.05$; $**p < 0.01$; $***p < 0.001$; $****p < 0.0001$.

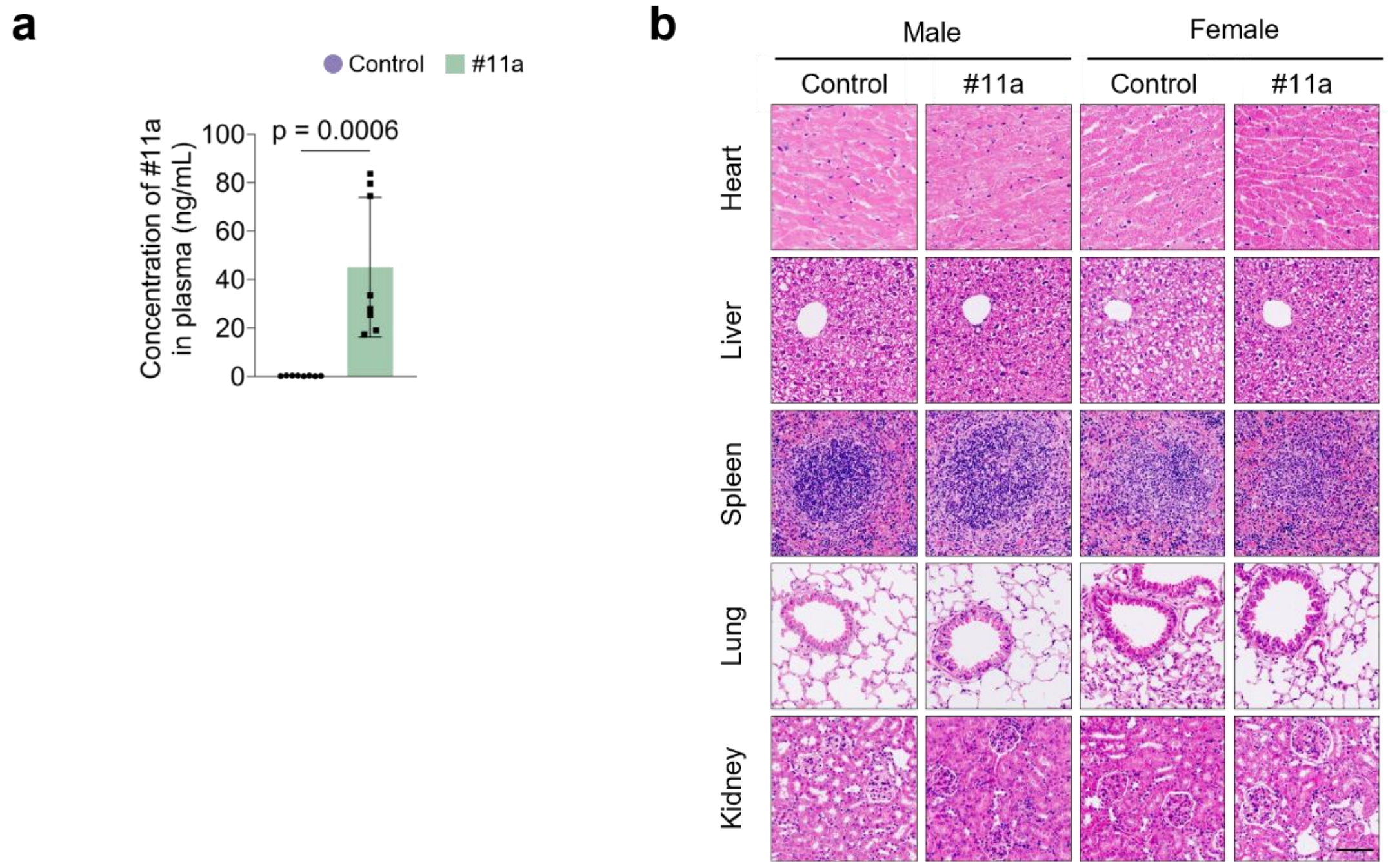

Figure S2. Pharmacokinetics and tissue histology.

(a) Concentration of #11a measured by LC-MS/MS after 5 months of #11a administration in serum from aged mice (n = 8). Data are presented as mean ± SD. Comparisons were performed using unpaired two-tailed Student's t tests. Significance levels are indicated as follows: *$p < 0.05$; **$p < 0.01$; ***$p < 0.001$; ****$p < 0.0001$. (b) Representative H&E staining images of heart, liver, spleen, lung, and kidney collected at 23 months of age. Scale bar, 50 μm.

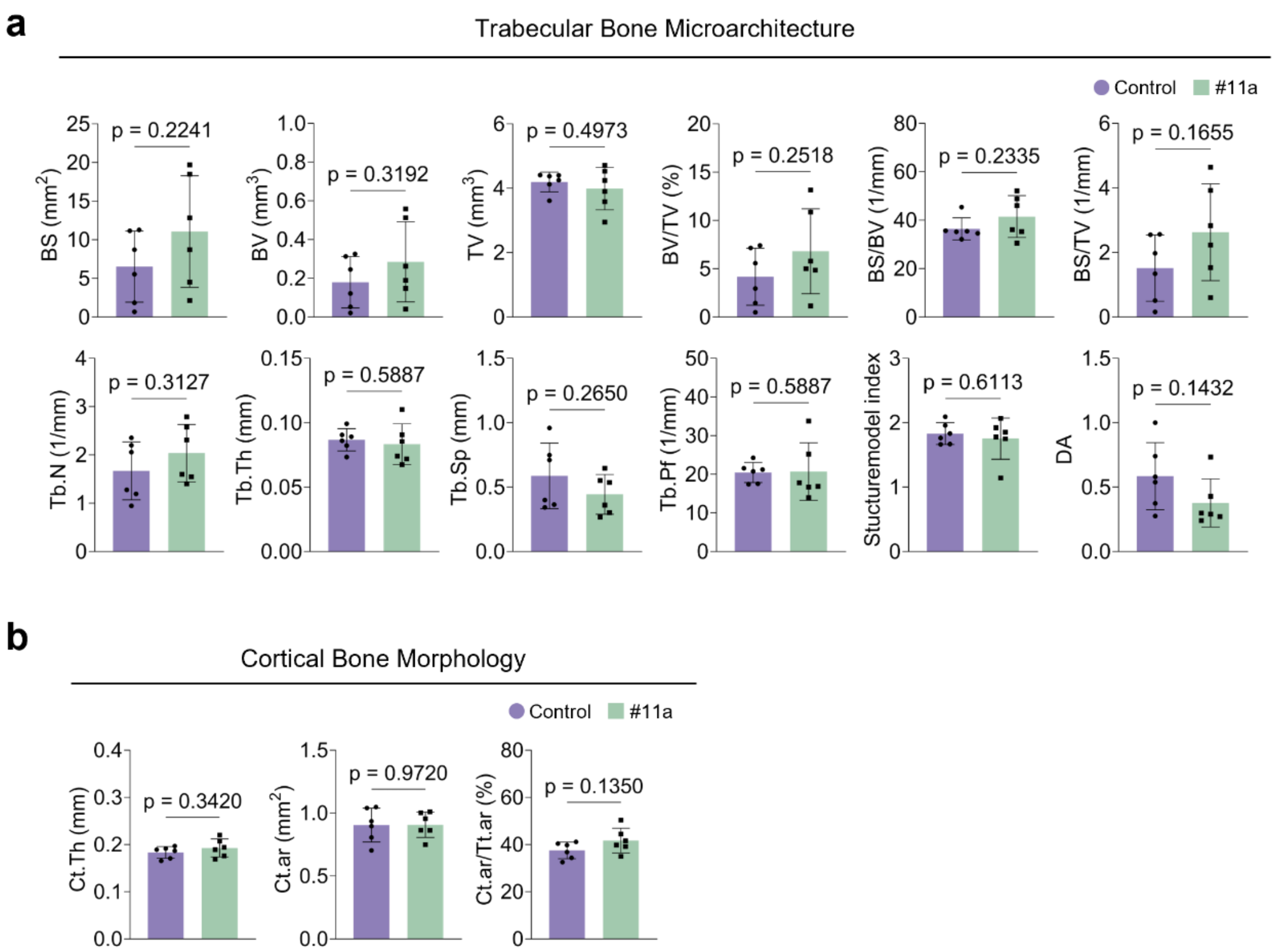

Figure S3. Echocardiographic and bone microarchitecture analysis.

(a) Trabecular bone microarchitecture parameters: bone surface (BS), bone volume fraction (BV/TV), total volume (TV), bone surface to bone volume ratio (BS/BV), bone surface to total volume ratio (BS/TV), structural model index (SMI), trabecular spacing (Tb.Sp), trabecular number (Tb.N), and trabecular thickness (Tb.Th). (b) Cortical bone morphological parameters: cortical thickness (Ct.Th) , cortical bone area (Ct.Ar), and the ratio of cortical bone area to total tissue area (Ct.Ar/Tt.Ar). n = 6. Data are presented as mean ± SD. Comparisons were performed using unpaired two-tailed Student's t tests; no statistically significant differences were observed in the parameters shown.

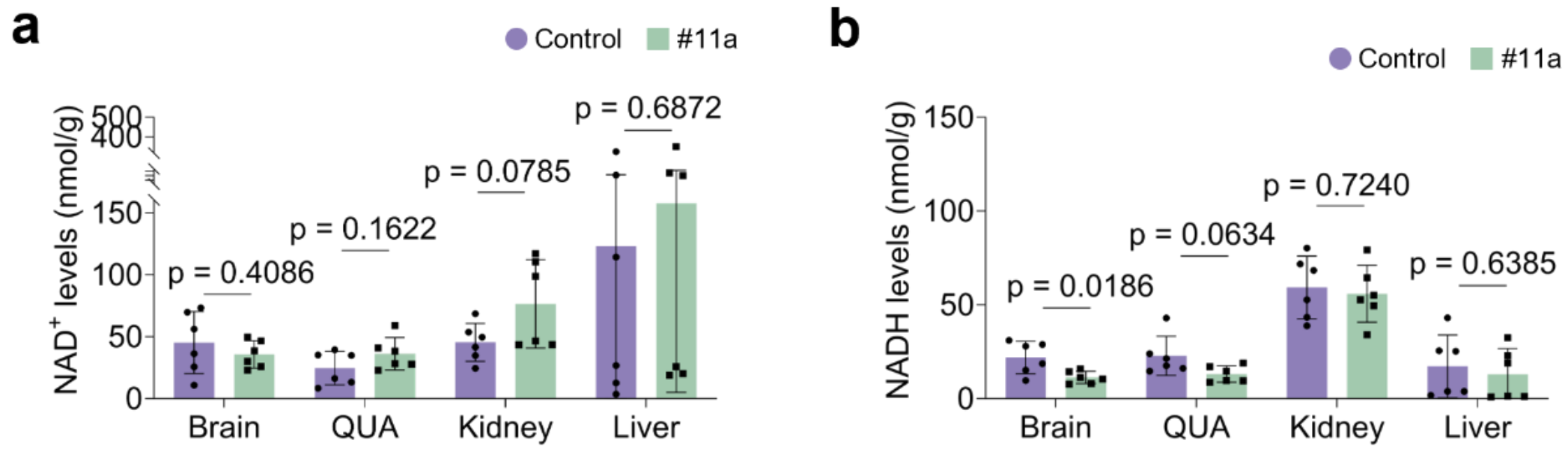

Figure S4. Effects of #11a on NAD$^+$/NADH-related measurements in tissues.

(a) NAD$^+$ levels in brain, quadriceps muscle, liver and kidney from 23-month-old mice (n = 6). (b) NADH levels in brain, quadriceps muscle, liver, and kidney from 23-month-old mice (n = 6). Data are presented as mean ± SD. Statistical significance was determined by unpaired two-tailed Student's t test. Significance levels are indicated as $^*p < 0.05$, $^{**}p < 0.01$, $^{***}p < 0.001$, $^{****}p < 0.0001$.

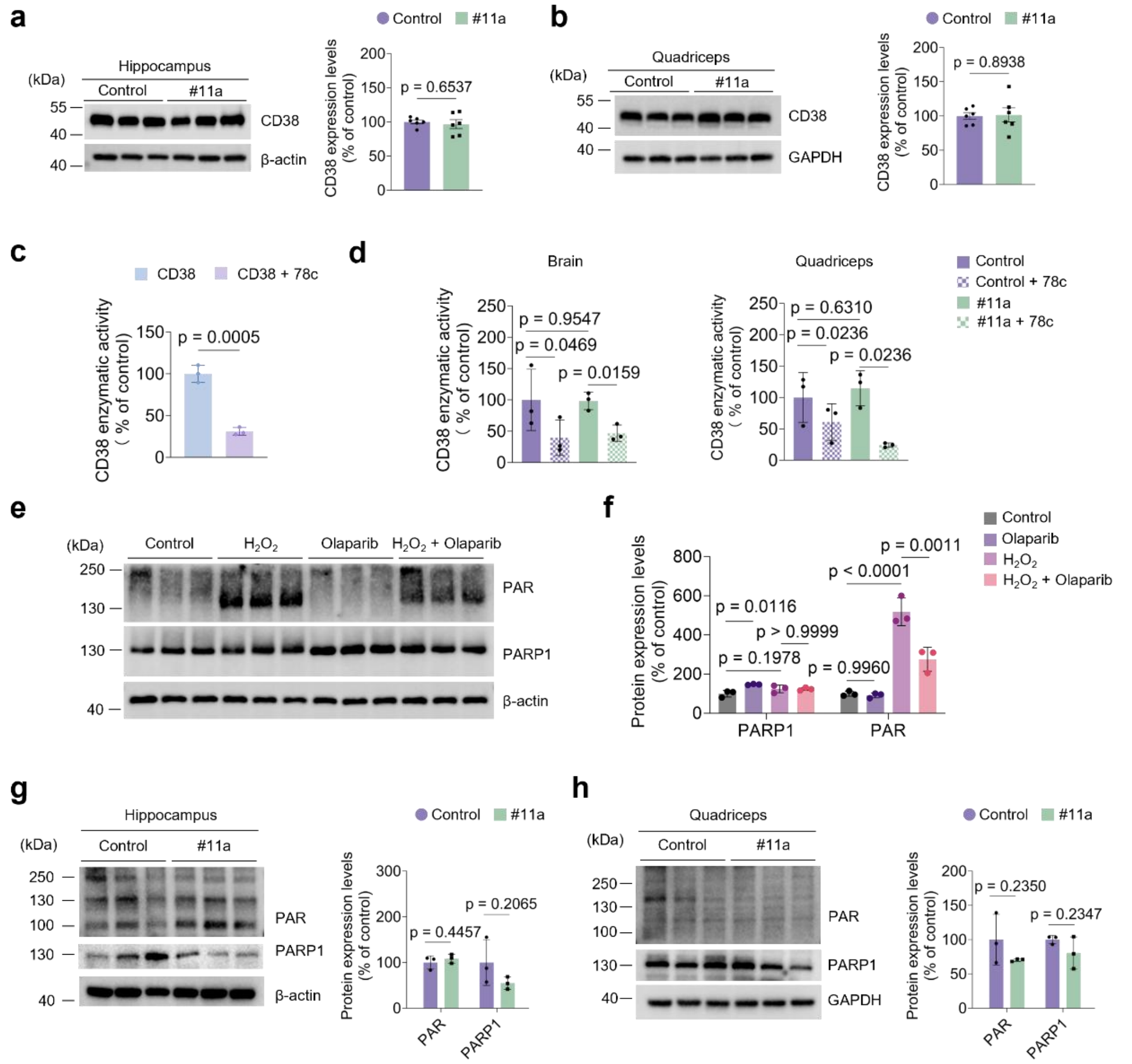

Figure S5. CD38 NADase activity and PARylation readouts.

(a-b) Immunoblot analysis and quantification of CD38 in hippocampus (a) and quadriceps (b) tissues. (c-d) CD38 NADase activity (ε-NAD hydrolysis) measured in recombinant human CD38 (c) and in hippocampus and quadriceps tissue lysates (d) in the presence or absence of 78c. (e-f) Technical positive control for anti-PAR immunoblotting in HT22 cells. H_2O_2 induced PAR smear formation, and olaparib reduced this signal. (g-h) Western blot analysis of global PARylation and PARP1 in hippocampus (g) and quadriceps (h) tissues from control and #11a-treated mice. Data are presented as mean ± SD; n = 3 independent samples per group unless indicated

otherwise. Statistical significance was determined by unpaired two-tailed Student's t test or ordinary one-way ANOVA with Bonferroni's multiple-comparison test as appropriate. Significance levels are indicated as *$p < 0.05$, **$p < 0.01$, ***$p < 0.001$, ****$p < 0.0001$.

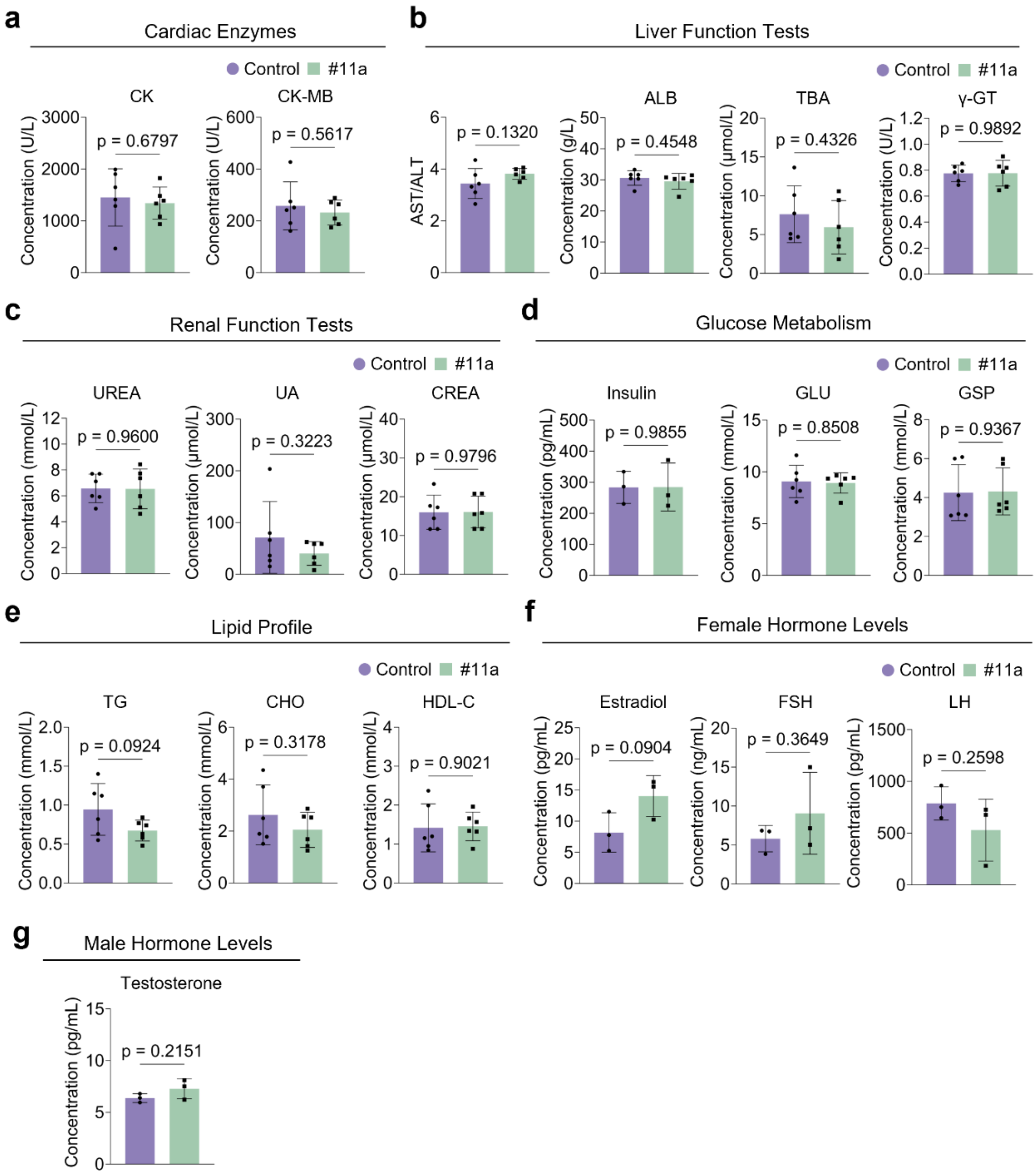

Figure S6. Serum biochemical and metabolic hormone profiles.

(a-e) Blood biochemistry tests from 23-month-old mice to examine cardiac enzyme (a), liver function (b), renal function (c), glucose metabolism (d) and blood lipid levels (e) related markers in serum (n = 6 per group). (f) Circulating reproductive hormones, including estradiol, follicle-stimulating hormone (FSH), and luteinizing hormone (LH), measured by ELISA from female mice (n = 3 per group). (g) Circulating reproductive hormone, including testosterone, measured by ELISA from male mice (n = 3 per group).

Data are presented as mean ± SD. Statistical significance was determined by unpaired two-tailed Student's t test. Significance levels are indicated as *$p < 0.05$, **$p < 0.01$, ***$p < 0.001$, ****$p < 0.0001$.

Table S1. Complete blood count

Parameter	Abbreviation	Result		Unit	p-Value
		Control	#11a		
White Blood Cell Count	WBC	5.19 ± 2.36	6.45 ± 3.20	^10^9/L	0.4025
Neutrophil Count (Absolute)	Neu#	1.43 ± 0.70	1.58 ± 0.80	^10^9/L	0.7508
Lymphocyte Count (Absolute)	Lym#	3.13 ± 1.64	4.17 ± 2.50	^10^9/L	0.4705
Monocyte Count (Absolute)	Mon#	0.51 ± 0.33	0.53 ± 0.32	^10^9/L	0.6859
Eosinophil Count (Absolute)	Eos#	0.11 ± 0.06	0.17 ± 0.09	^10^9/L	0.0827
Basophil Count (Absolute)	Bas#	0.00 ± 0.01	0.01 ± 0.01	^10^9/L	0.6512
Neutrophil Percentage	Neu%	29.48 ± 10.13	25.53 ± 8.47	%	0.4188
Lymphocyte Percentage	Lym%	58.54 ± 11.63	63.01 ± 12.39	%	0.2726
Monocyte Percentage	Mon%	9.72 ± 4.35	8.65 ± 4.12	%	0.4703
Eosinophil Percentage	Eos%	2.13 ± 0.61	2.70 ± 0.82	%	0.0777
Basophil Percentage	Bas%	0.13 ± 0.08	0.11 ± 0.12	%	0.3187
Red Blood Cell Count	RBC	8.03 ± 0.95	7.92 ± 1.41	^10^{12}/L	0.9310
Hemoglobin	HGB	125.50 ± 8.69	125.17 ± 19.91	g/L	0.3859
Hematocrit	HCT	42.73 ± 2.56	41.99 ± 5.84	%	0.8852
Mean Corpuscular Volume	MCV	53.84 ± 6.05	53.84 ± 5.94	fL	0.9310
Mean Corpuscular Hemoglobin	MCH	15.78 ± 1.46	15.93 ± 1.03	pg	0.9539
Mean Corpuscular Hemoglobin Concentration	MCHC	293.67 ± 7.58	296.92 ± 14.72	g/L	0.1328
Red Cell Distribution Width - Coefficient of Variation	RDW-CV	19.40 ± 3.51	17.56 ± 1.96	%	0.1407
Red Cell Distribution Width - Standard Deviation	RDW-SD	40.59 ± 9.34	37.31 ± 7.75	fL	0.1748
Platelet Count	PLT	1563.83 ± 645.98	1691.17 ± 569.90	^10^9/L	0.5444
Mean Platelet	MPV	7.58 ± 0.31	7.38 ± 0.57	fL	0.3846

Volume					
Platelet Distribution Width	PDW	15.47 ± 0.32	15.48 ± 0.25		0.8383
Plateletcrit	PCT	0.79 ± 0.19	0.89 ± 0.05	%	0.7842

Complete blood count parameters in 23-month-old mice treated with #11a or control diet from 18 months of age (n = 12 per group). All data are presented as the mean ± SD. Statistical comparisons between the control and #11a groups were performed using unpaired two-tailed Student's t tests. Exact p values are shown in the table.

DATA S1: Uncropped blot and gel images for Figure 2l

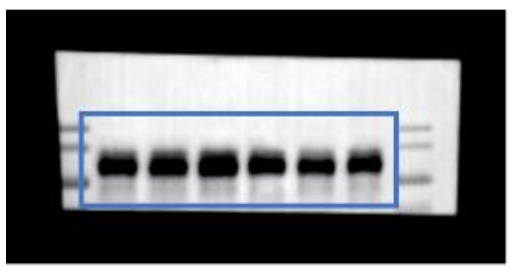

Anti-APP N

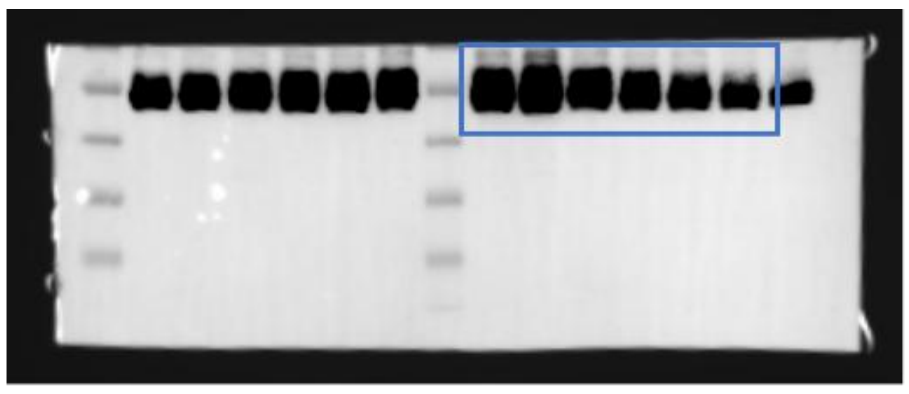

Anti-Tau 5

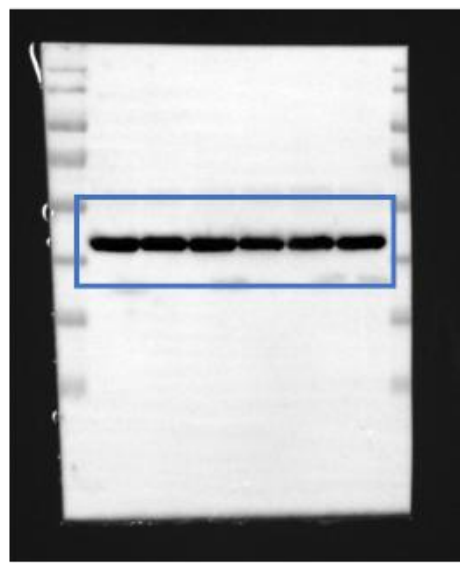

Anti-β-actin

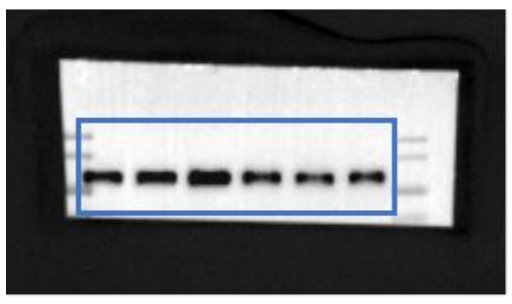

Anti-APP N585

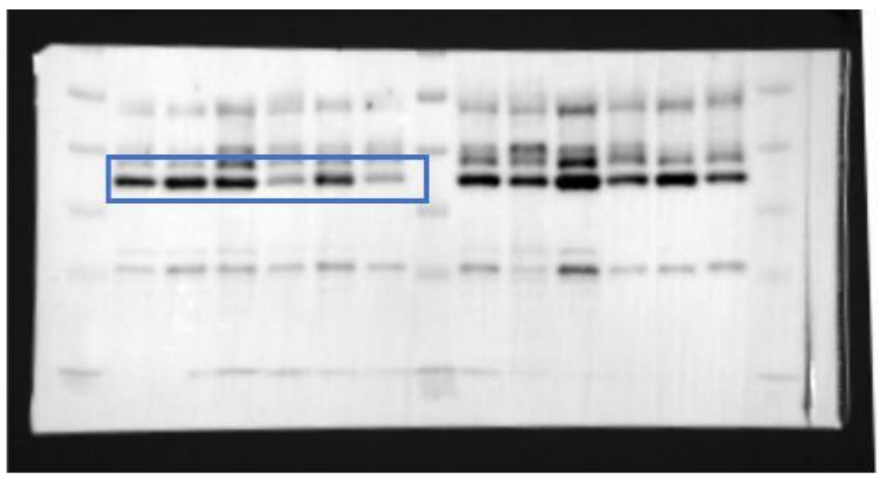

Anti-Tau N368

DATA S1: Uncropped blot and gel images for Figure 3a

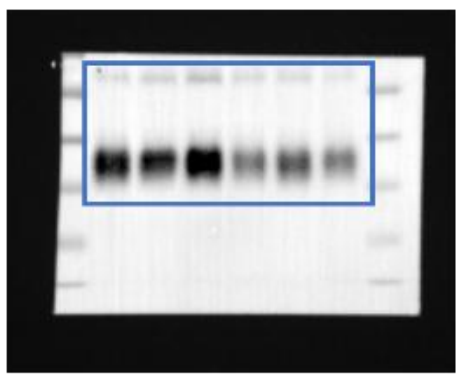
Anti-AEP

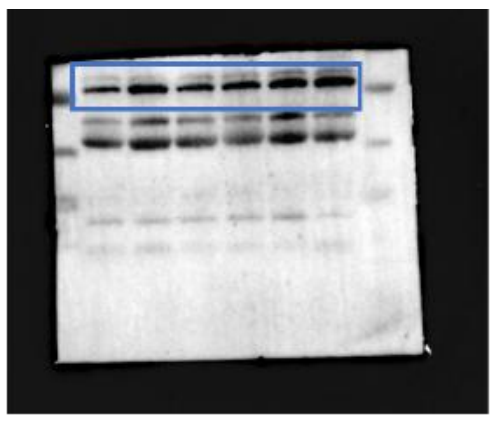
Anti-NAMPT

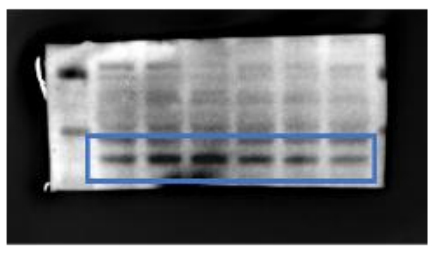
Anti-NAMPT C137

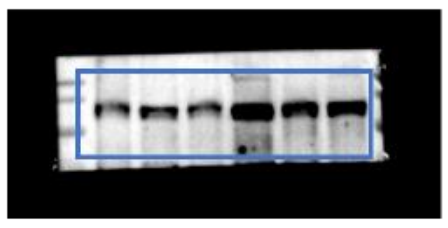
Anti- SIRT1

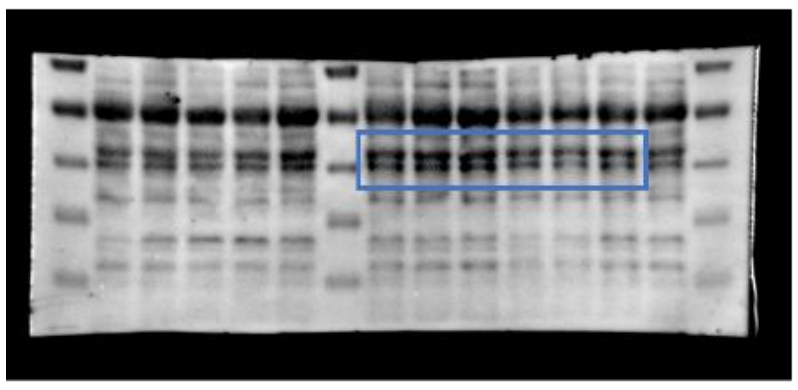
Anti-C/EBPβ

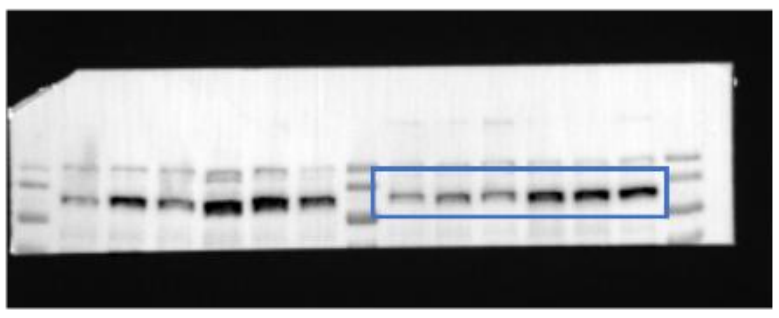
Anti- PGC-1α

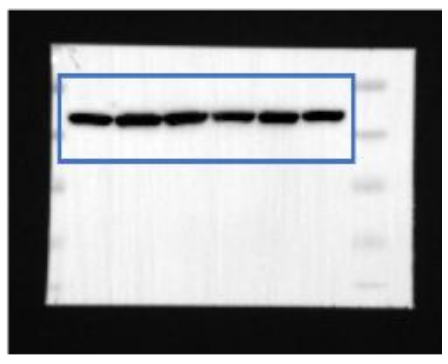
Anti- β-actin

DATA S1: Uncropped blot and gel images for Figure 3f

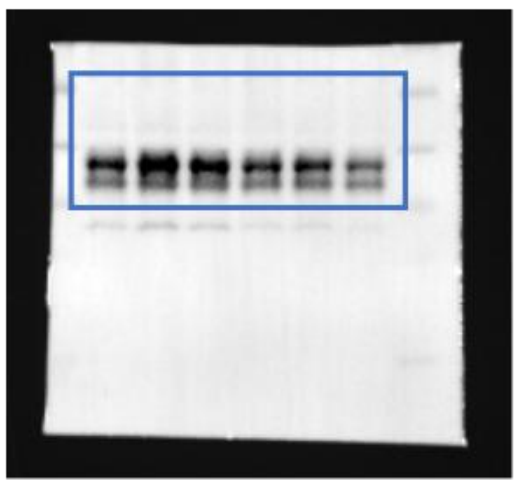
Anti-AEP

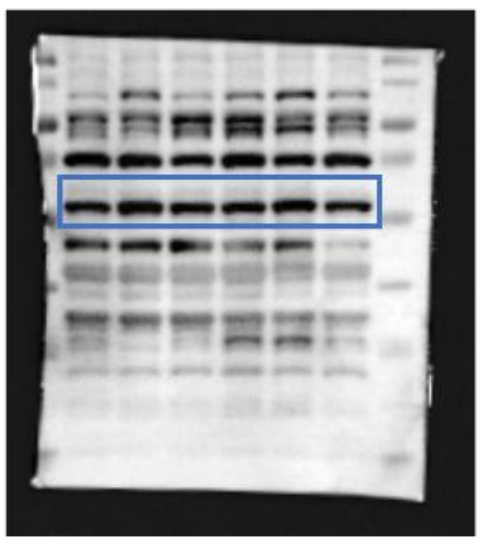
Anti-NAMPT

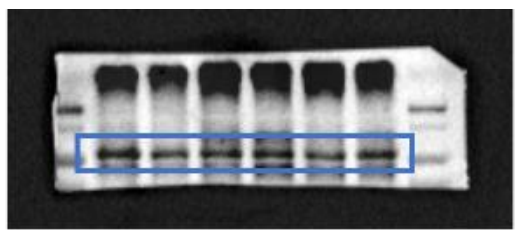
Anti-SIRT1

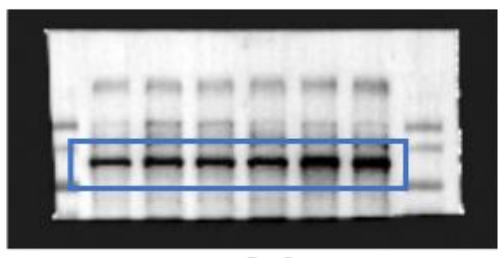
Anti-PGC-1α

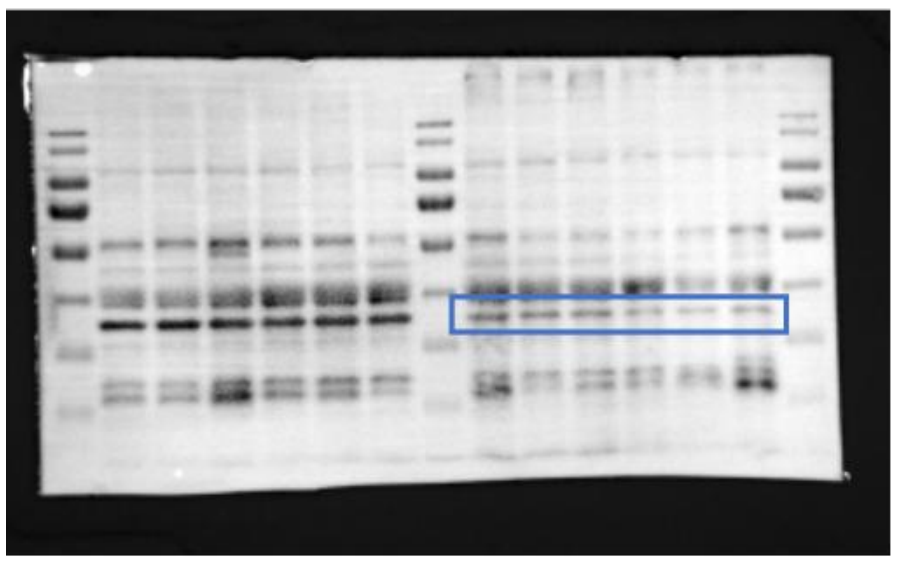
Anti-C/EBPβ

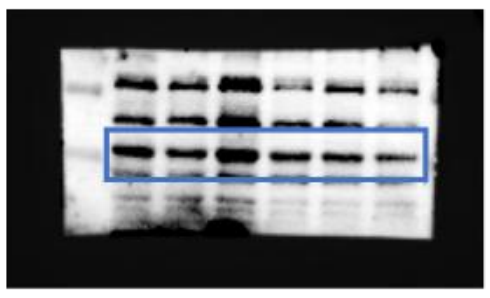
Anti-NAMPT C137

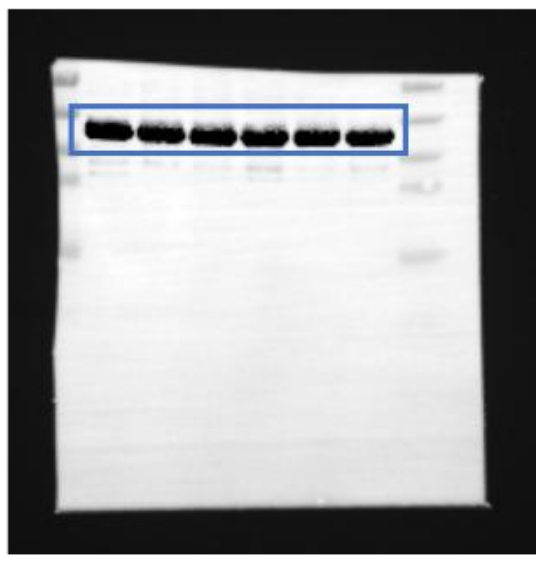
Anti-GAPDH

DATA S1: Uncropped blot and gel images for Figure 5c liver

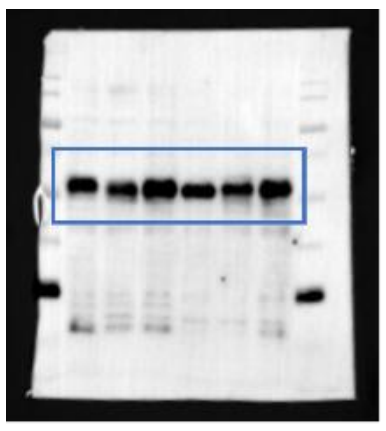

Anti-p53

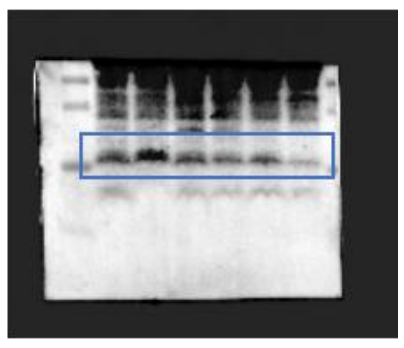

Anti-p16

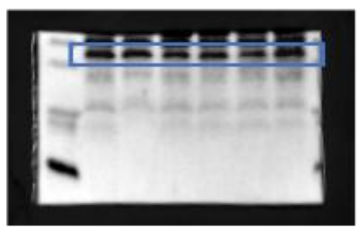

Anti-p21

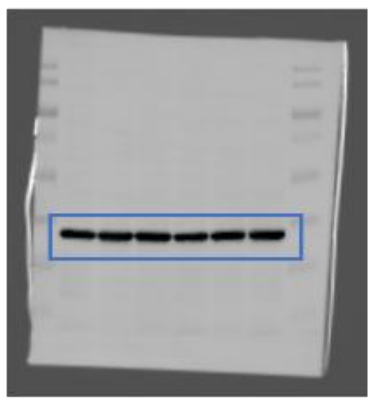

Anti-GAPDH

DATA S1: Uncropped blot and gel images for Figure 5c kidney

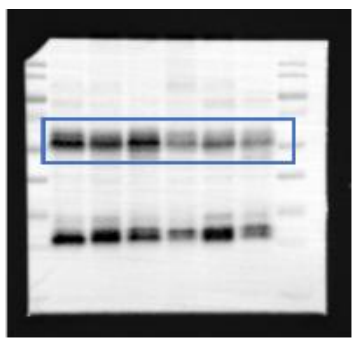

Anti-p53

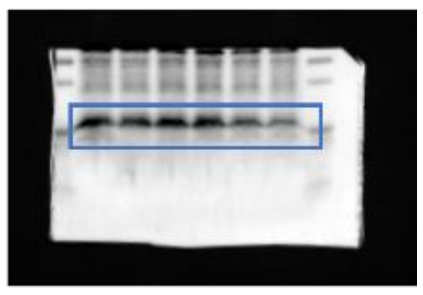

Anti-p16

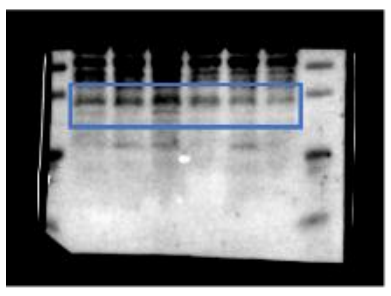

Anti-p21

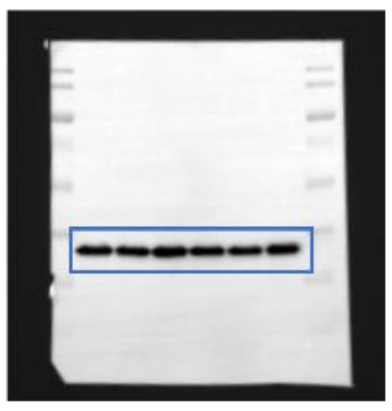

Anti-GAPDH

DATA S1: Uncropped blot and gel images for Figure 6b

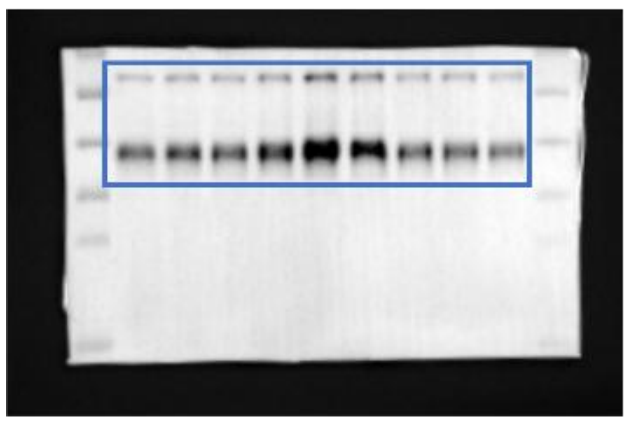

Anti-AEP

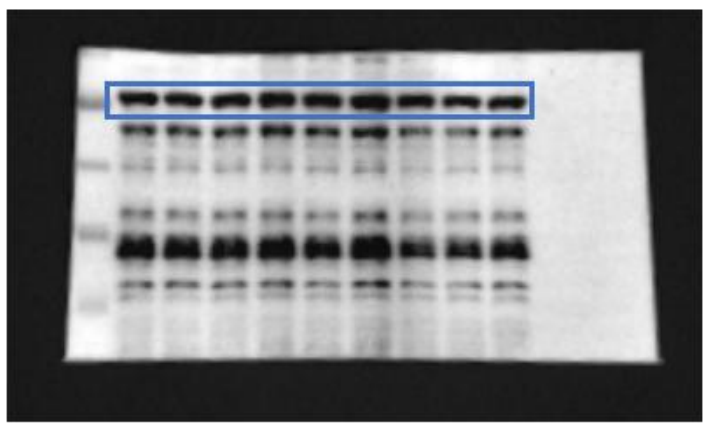

Anti-NAMPT

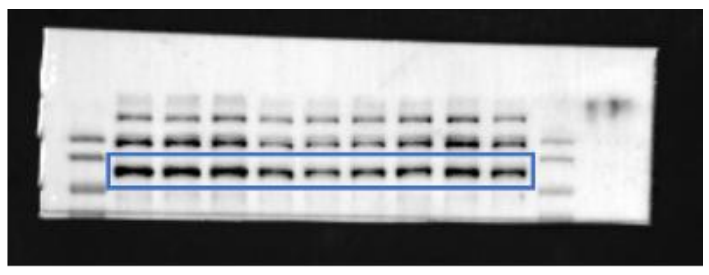

Anti-PGC-1α

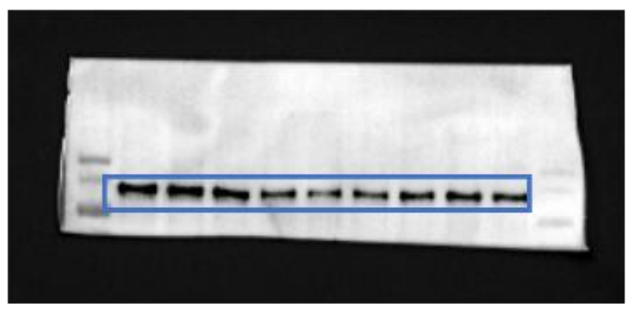

Anti-SIRT1

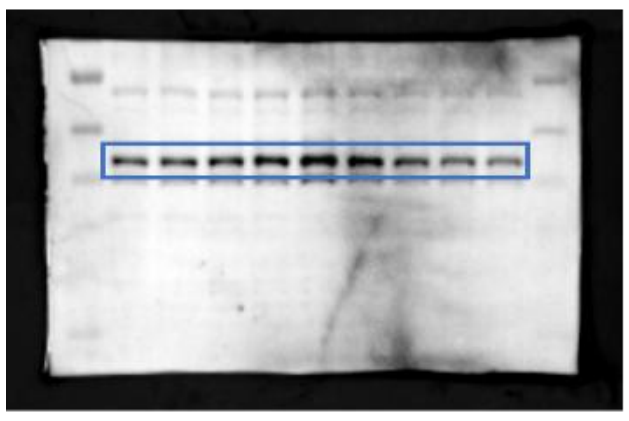

Anti-C/EBPβ

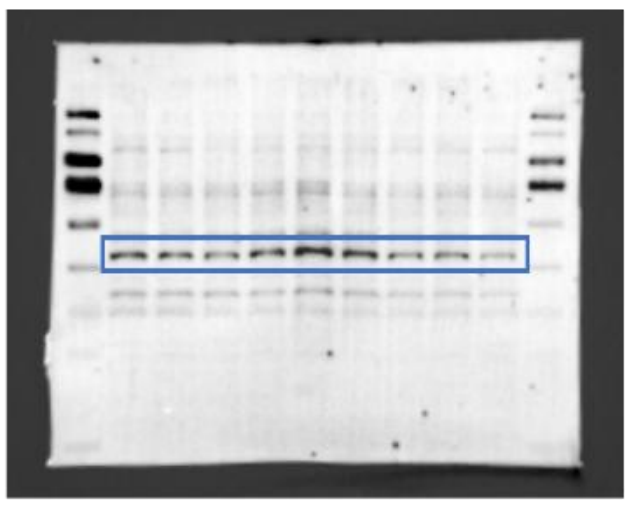

Anti-NAMPT C137

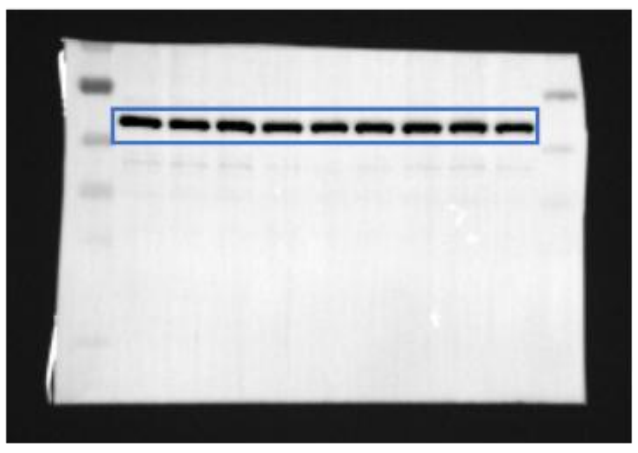

Anti-β-actin

DATA S1: Uncropped blot and gel images for Figure 6g

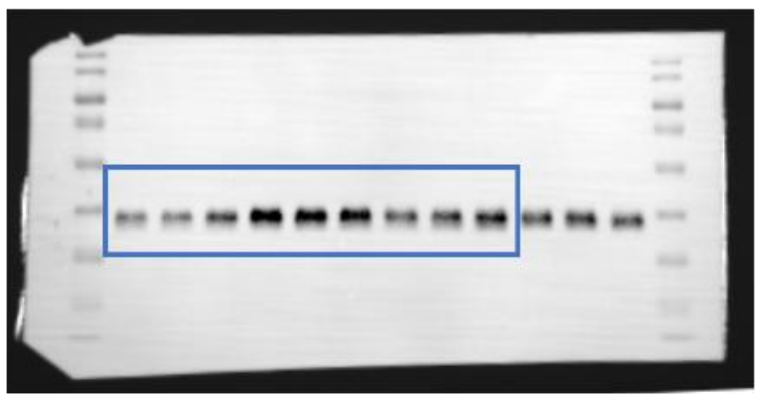

Anti-AEP(Short exposure)

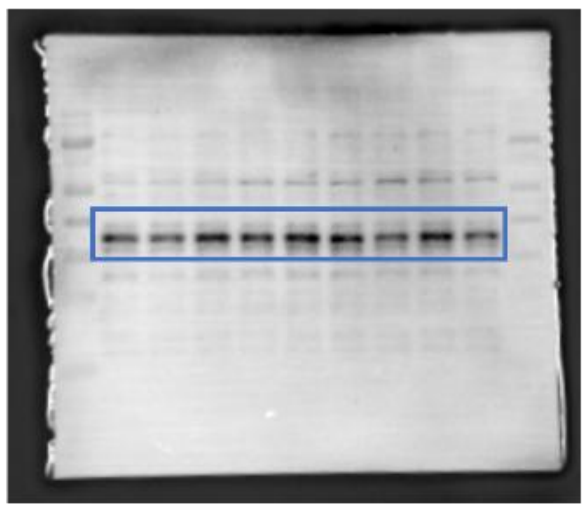

Anti-C/EBPβ

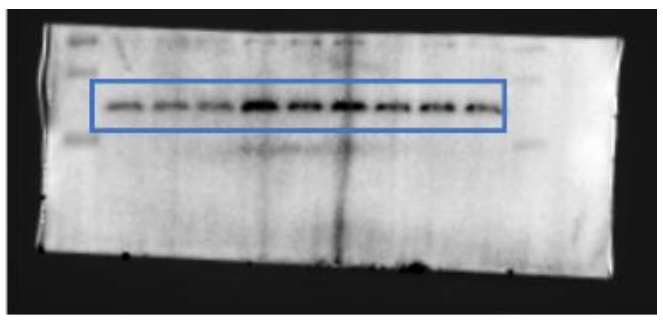

Anti-p21

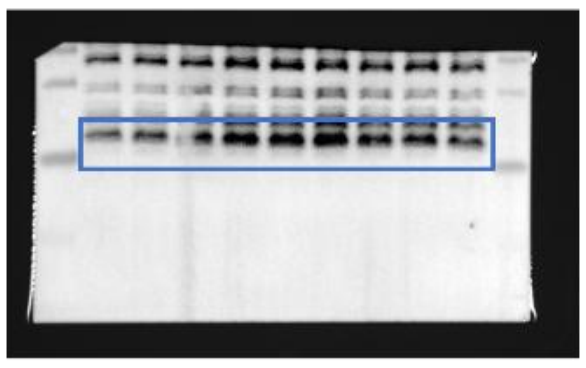

Anti-p16

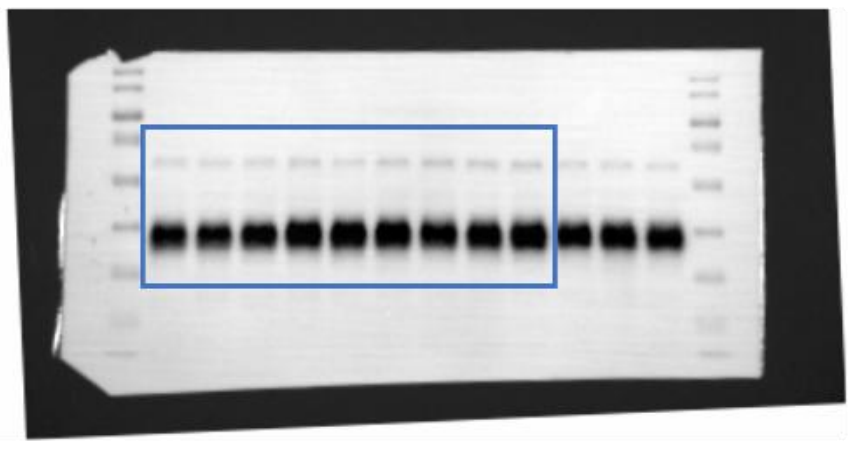

Anti-AEP(Long exposure)

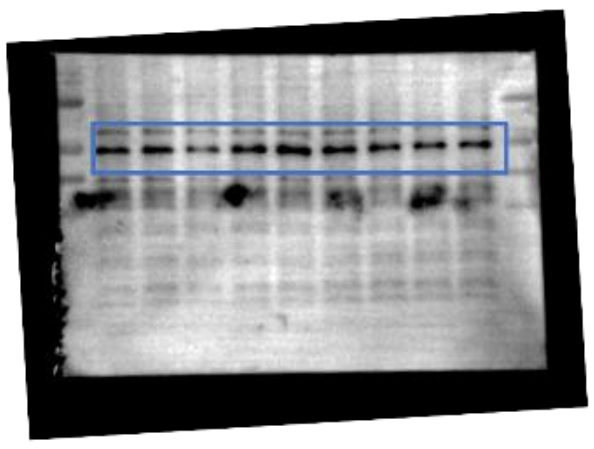

Anti-p53

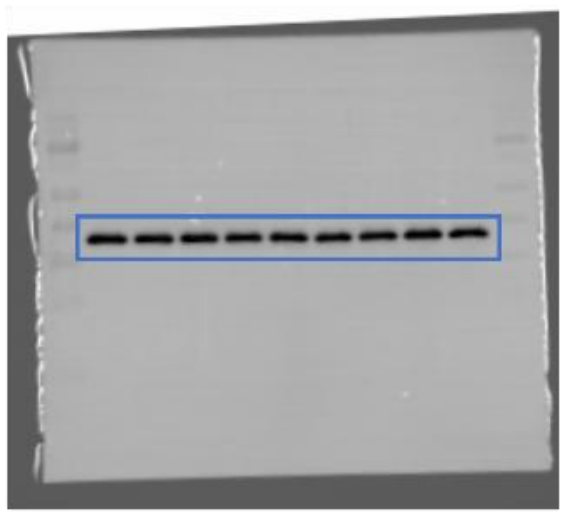

Anti-GAPDH

DATA S1: Uncropped blot and gel images for Figure S5a&S5b

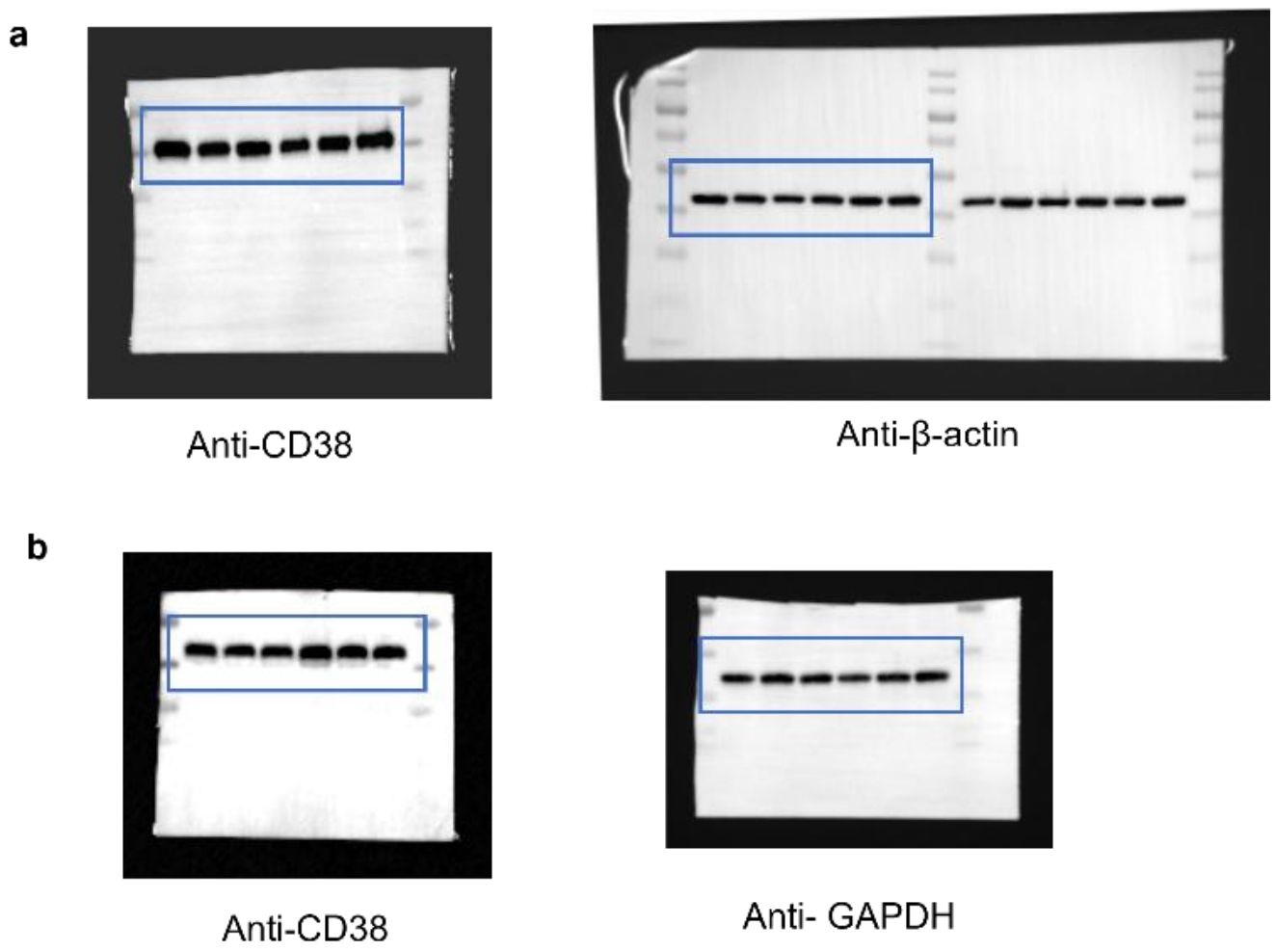

DATA S1: Uncropped blot and gel images for Figure S5e

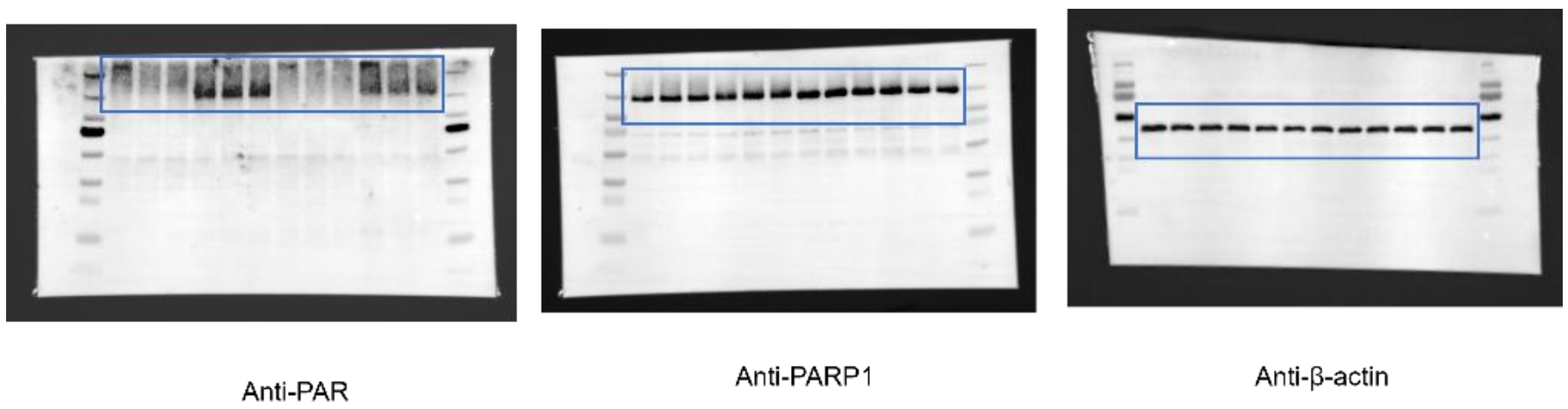

DATA S1: Uncropped blot and gel images for Figure S5g&S5h

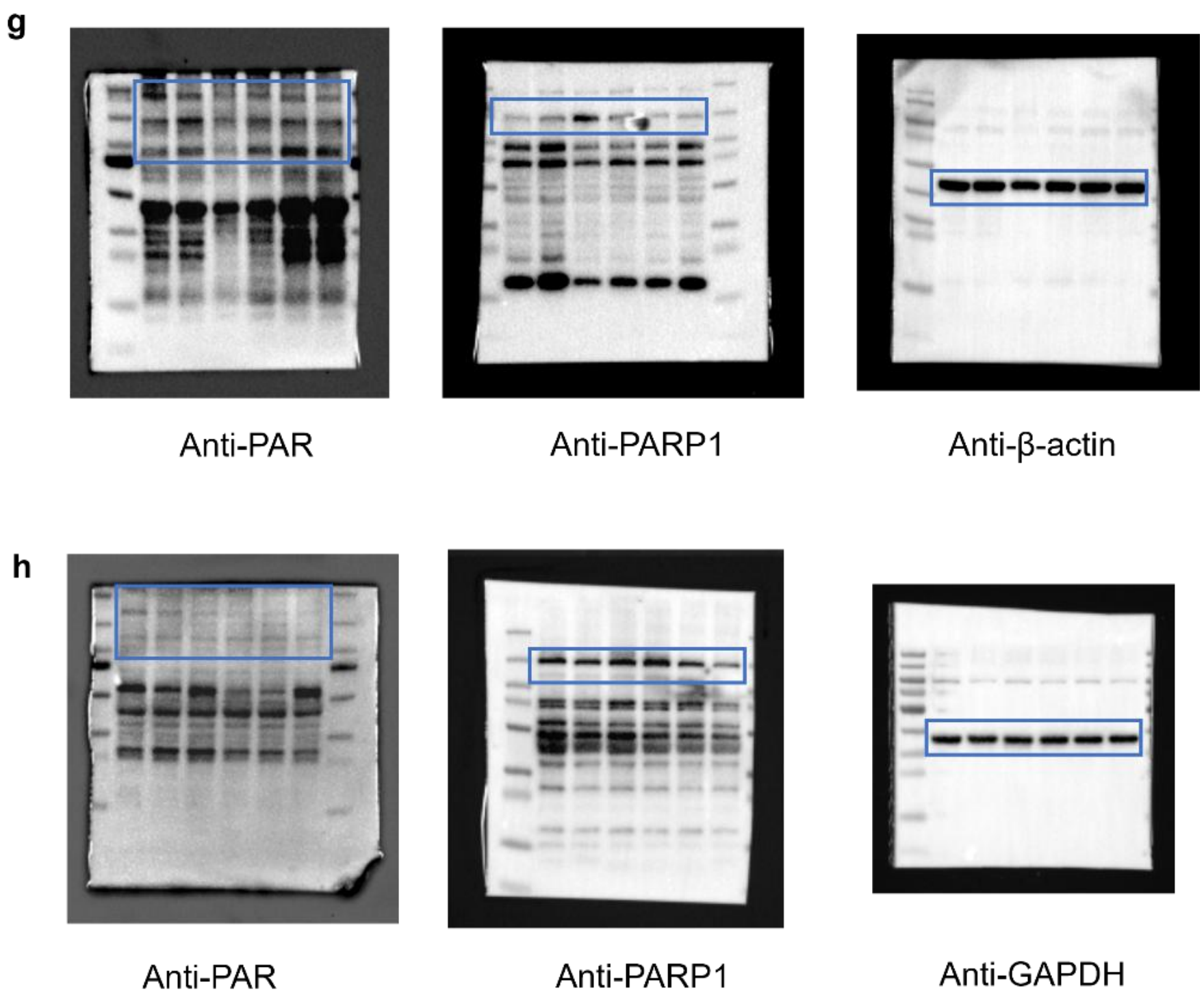

Supplement: Supplementary file 1 [file mmc1.pdf]
